# Temperature-induced microstructural changes in shells of laboratory-grown *Arctica islandica* (Bivalvia)

**DOI:** 10.1371/journal.pone.0247968

**Published:** 2021-02-26

**Authors:** Nils Höche, Eric O. Walliser, Niels J. de Winter, Rob Witbaard, Bernd R. Schöne

**Affiliations:** 1 Institute of Geosciences, University of Mainz, Mainz, Germany; 2 Department of Earth Sciences, Faculty of Geosciences, Utrecht University, Utrecht, The Netherlands; 3 AMGC Research Group, Vrije Universiteit Brussel, Brussels, Belgium; 4 Department of Estuarine & Delta Systems, NIOZ Royal Netherlands Institute for Sea Research, Yerseke, The Netherlands; Union College, UNITED STATES

## Abstract

Bivalve shells are increasingly used as archives for high-resolution paleoclimate analyses. However, there is still an urgent need for quantitative temperature proxies that work without knowledge of the water chemistry–as is required for δ^18^O-based paleothermometry–and can better withstand diagenetic overprint. Recently, microstructural properties have been identified as a potential candidate fulfilling these requirements. So far, only few different microstructure categories (nacreous, prismatic and crossed-lamellar) of some short-lived species have been studied in detail, and in all such studies, the size and/or shape of individual biomineral units was found to increase with water temperature. Here, we explore whether the same applies to properties of the crossed-acicular microstructure in the hinge plate of *Arctica islandica*, the microstructurally most uniform shell portion in this species. In order to focus solely on the effect of temperature on microstructural properties, this study uses bivalves that grew their shells under controlled temperature conditions (1, 3, 6, 9, 12 and 15°C) in the laboratory. With increasing temperature, the size of the largest individual biomineral units and the relative proportion of shell occupied by the crystalline phase increased. The size of the largest pores, a specific microstructural feature of *A*. *islandica*, whose potential role in biomineralization is discussed here, increased exponentially with culturing temperature. This study employs scanning electron microscopy in combination with automated image processing software, including an innovative machine learning–based image segmentation method. The new method greatly facilitates the recognition of microstructural entities and enables a faster and more reliable microstructural analysis than previously used techniques. Results of this study establish the new microstructural temperature proxy in the crossed-acicular microstructures of *A*. *islandica* and point to an overarching control mechanism of temperature on the micrometer-scale architecture of bivalve shells across species boundaries.

## Introduction

Seasonally to annually resolved past ocean temperature data are crucial to better understand feedbacks in the global climate system and constrain climate simulations [1–3]. This type of information is increasingly reconstructed from bivalve shells [4–6], specifically stable oxygen isotope (δ^18^O_shell_) values [7–9]. Given known limitations of this paleothermometer, i.e., vulnerability to diagenetic overprint [10] and difficulties to obtain δ^18^O_water_ signatures of ancient and/or coastal water bodies, the search for alternative temperature proxies is in full swing (Δ_47_: [11, 12]; Δ_48_: [13]; Mg, Sr: [14]). A highly promising candidate is the shell microstructure [15–18]. It cannot only provide sub-seasonally resolved temperature data, but is also much less susceptible to diagenetic overprint than geochemical properties of shells [19, 20] and, contrary to the incorporation of trace and minor elements into the shells [21–23], appears to be unaffected by kinetic and vital effects [24, 25].

**Fig 1:**
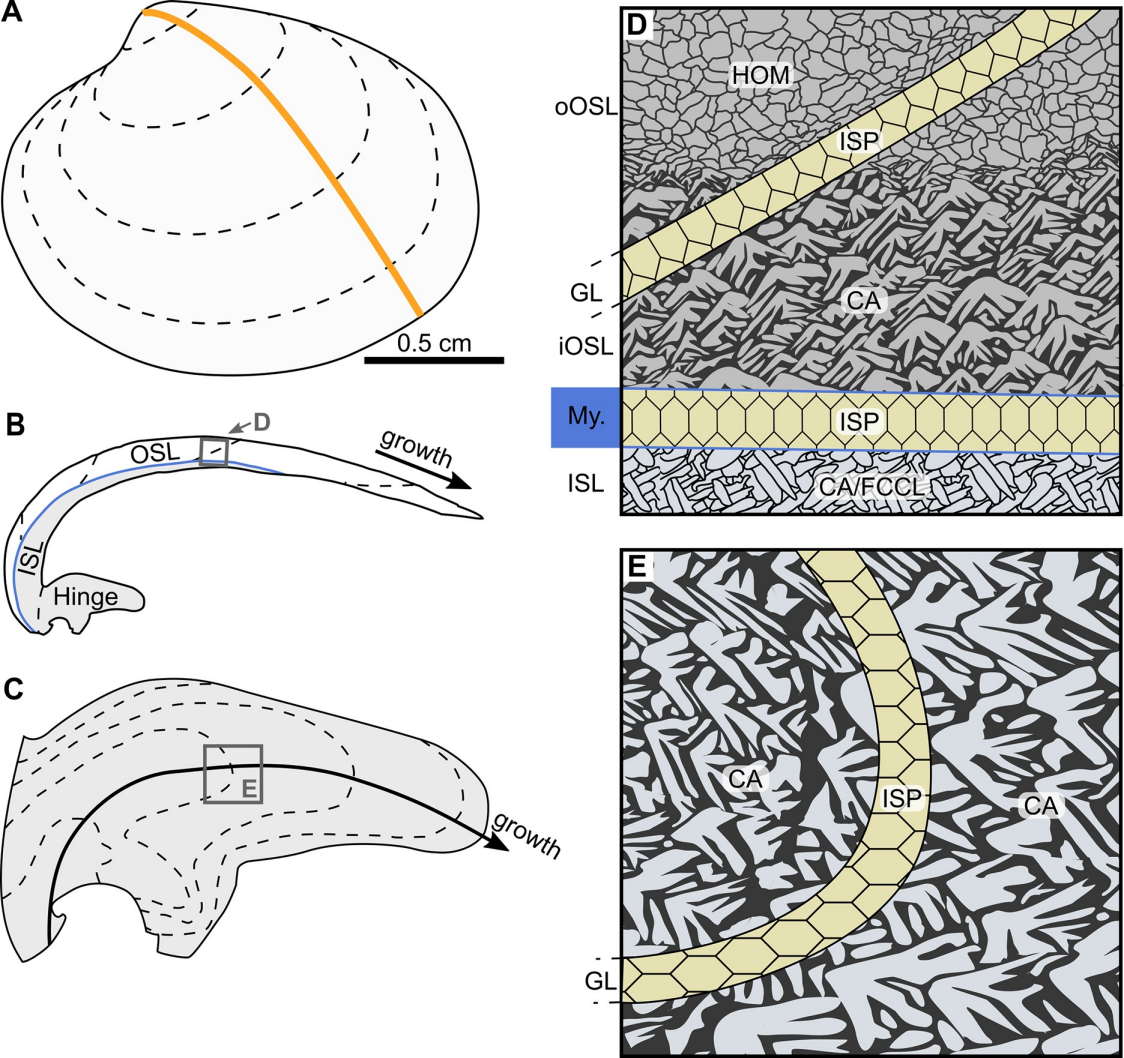
Shell growth patterns and microstructure of *Arctica islandica*. (A) Sketch of the left valve of a juvenile specimen. Cutting axis is indicated as orange line. (B) Radial shell section of the valve. The shell is divided into an outer shell layer (OSL; white) and inner shell layer (ISL; gray), separated by the pallial myostracum (blue line). (C) Magnified sketch of the hinge plate showing annual growth lines (dashed) and axis of maximum growth (black line with arrow). (D) Sketch of the microstructures of the ventral shell portion. The outer portion of the outer shell layer (oOSL) consists of homogeneous (HOM) microstructure, which gradually merge into crossed-acicular (CA) microstructure toward the inner portion of the outer shell layer (iOSL). Transitional fine complex crossed-lamellar (FCCL) and CA microstructures are formed in the inner shell layer (ISL). Annual growth lines (GL) and pallial myostracum (My.) consist of irregular simple prismatic (ISP) microstructure. (E) Sketch of the microstructures in the hinge plate. Growth increments and lines are composed of CA and ISP microstructures, respectively. Dashed black lines represent annual growth lines. Boxes in B and C show the extent of the microstructure sketches portrayed in D and E, respectively.

As demonstrated by existing studies, increasingly larger and/or more elongated biomineral units (BMUs) are formed when temperature rises. This applies to bivalves with nacreous (*Pinna* sp. and *Atrina* sp.: [24]), prismatic (*Cerastoderma edule*: [25]) and crossed-lamellar microstructures (*Glycymeris bimaculata*: [26]). However, in the long-lived ocean quahog, *Arctica islandica*, Milano et al. [27] were unable to identify temperature-related morphological changes of individual BMUs. The reservation must be made that these authors only evaluated the microstructures in the ventral margin by visual assessment, while placing the focus of their study on the relationship between the crystallographic orientation of BMUs and environmental variables. Due to the enormous microstructural diversity–in particular in shell portions of the ventral margin–morphometric analysis of BMUs in *A*. *islandica* is a particularly challenging task. For example, the homogeneous microstructure (HOM) in the outer portion of the outer shell layer of *A*. *islandica* gradually merges into crossed-acicular (CA) microstructure toward the myostracum (Fig 1A–1D) [27, 28]. In addition, the thickness of the HOM portion increases with ontogenetic age [29]. Yet, fine complex crossed-lamellar (FCCL) microstructure occurs in the inner portion of the outer shell layer and in the inner shell layer (ISL) [28, 30]. As in almost all other mollusks, the annual growth lines are dominated by irregular simple prismatic microstructures (ISP) [28, 31, 32]. The only region in *A*. *islandica* which is microstructurally relatively uniform is the hinge (growth increments: predominantly CA; annual growth lines: ISP) [28, 33, 34]. Given the important role of the ocean quahog in sclerochronology-based paleoclimate reconstructions [4, 35–37] including the more distant past [38], the known limitations of geochemical proxies outlined above and promising results in recent BMU studies, a more detailed assessment of the shell microstructure of *A*. *islandica* as a possible recorder of water temperature seems overdue.

Here we investigate possible effects of temperature on microstructural properties in the hinge plate of young *A*. *islandica* specimens. To minimize interferences with other environmental variables and thus following the rationale of Milano et al. [27], we studied shell portions that grew under controlled laboratory conditions. Our study is based on 17 specimens that were cultured for 95 days at six different temperature regimes (1° to 15°C; Table 1). Machine learning–assisted image segmentation was used to (reproducibly and objectively) identify individual BMUs and μm-sized pores, a characteristic feature in *A*. *islandica* [28, 30, 39], in scanning electron microscopy (SEM) images of polished shell cross-sections after immersion in H_2_O_2_. The size (area) of individual BMUs and pores was automatically determined by means of image processing software. Since the individual BMUs of the CA microstructure are often challenging to distinguish, even for currently available user-trained artificial intelligence software, the proportion covered by the BMUs (= BMU coverage) was also determined. The new technique presented herein not only provides more robust data than manual microstructure analyses and visual inspection, but also vastly accelerates the measurements compared with previously used image processing techniques. Our approach can pave the way toward improved high-resolution paleotemperature estimates from bivalve shells, specifically *A*. *islandica*.

**Table 1:** Shells of *Arctica islandica* used in the present study.

Specimen ID: “Nioz-TC-…”	Specimen ID in original culturing study^a^	# LA-ICP-MS measurements	BMU morphometry (μm^2^)	Pore morphometry (μm^2^)	Culturing temperature (°C)
01-A1R	89	16	7,320	2,455	1.1 ± 0.2
01-A2R^b^	71	19	1,220	720	
03-A1R	69	19	13,420	10,977	3.2 ± 0.4
03-A2R	36	23	3,660	22,555	
03-A3R	20	-	10,980	20,590	
06-A1R	55	29	14,640	23,274	6.2 ± 0.5
06-A2R	32	23	3,660	39,836	
06-A3R	21	22	10,980	33,764	
09-A1R	113	-	13,420	6,501	9.2 ± 0.6
09-A2R	98	21	6,100	10,541	
09-A3R	58	22	4,880	9,797	
12-A1R	149	-	1,220	13,505	12.0 ± 0.8
12-A2R	70	24	8,540	47,779	
12-A3R	44	21	10,980	27,566	
15-A1R	99	-	12,200	46,883	15.1 ± 0.4
15-A2R	93	-	1,220	36,789	
15-A3R	12	-	4,880	47,422	

^a^ Consecutive numbers were assigned to each shell in the original culturing study [40]. Here, new specimen IDs were assigned (given in leftmost column, all starting with “Nioz-TC-..”) to ease identification.

^b^ Due to the very limited number of samples remaining from this culturing experiment, only two specimens cultured at 1°C could be used.

## Material and methods

Shells of seventeen juvenile (five years-old) *A*. *islandica* specimens were used in the present study. Bivalves were collected alive on 8 February 1993 from the trawl site ‘Süderfahrt/Millionenviertel’, Kiel Bay, Germany, western Baltic Sea (54° 52’59”N, 010°08’00”E), at a water depth of 20 m using a “Kieler Kinderwagen” dredge and subsequently used in a culturing experiment [40]. After collection, bivalves were cooled, aerated and transferred to the Royal Netherlands Institute for Sea Research, Texel (The Netherlands), where they were distributed over different sand-filled containers supplied with filtered, constantly aerated seawater and acclimatized to North Sea conditions. After four weeks, the experimental aquaria were adjusted to constant temperature regimes (1°, 3°, 6°, 9°, 12°, and 15°C; Table 1) for 95 days. Food availability was kept *ad libitum* by supplying a (freshly cultured) phytoplankton mixture of *Isochrysis galbana* and *Dunaliella marina* with cell densities maintained in the optimum range of 10–20 × 10^6^ cells per liter [40, 41].

### Shell preparation

The right valve of each specimen was affixed to acrylic glass cubes with a quick-drying plastic welder (WIKO Multi Power 03) and all surfaces along the axis of growth from the umbo to the ventral margin covered with a ca. 1 cm broad and several mm-thick protective layer of metal epoxy resin (WIKO 05 epoxy). After curing, each valve was cut along the axis of maximum growth using a low-speed saw (Buehler IsoMet 1000) operated at 250 rpm and equipped with a diamond-coated wafering thin blade (Buehler 15LC 11–4255, 0.4 mm thickness, low-diamond concentration). From that axis, two shell slabs (2.5–3.0 mm thick) were obtained and embedded in Araldite 2020 mixed with conductive filler (Buehler 20–8500). All slabs were ground successively with P800, P1200 and P2500 grit SiC paper and then polished with 1.0 and 0.3 μm Al_2_O_3_ suspension using polishing cloths (Buehler MasterTex) on a rotating lap (Buehler MetaServ 2000) at 100 rpm. After each grinding and polishing step, samples were ultrasonically rinsed in tap water for two minutes. The final rinsing step was conducted with demineralized water to avoid precipitation of calcium carbonate impurities on the cross-sectioned surfaces. One shell slab of each specimen was used for trace element and growth pattern analyses and the other one for SEM studies.

### Identification of laboratory-grown shell portions

The laboratory grown shell portions appeared to be separated from the remainder of the shell by a distinct growth check (accompanied by a change of periostracum color) that was visible at first glance in marginal shell portions on the external surfaces of all shells. In order to confirm which shell portions of the hinge plates formed under controlled laboratory conditions, a combined geochemical, microstructural and growth pattern analysis was applied. The trace and minor element composition of bivalve shells can serve as a provenance indicator, because the water chemistry of the environment is partially reflected in the shells [42–45]. In particular, as experimentally demonstrated multiple times, elevated levels of dissolved manganese in the water are mirrored in the shell [e.g., 46, 47]. Since Mn is a redox-sensitive element, whose concentration increases with decreasing content of dissolved oxygen [48–50], bivalves living in low-oxygen environments such as settings below the seasonal halocline in the Baltic Sea [e.g., 51–54] show higher Mn concentrations than specimens from well-oxygenated waters (own observation). Shell portions formed in tanks supplied with oxygen-rich North Sea water are thus expected to show significantly lower Mn/Ca values than those grown in the Baltic Sea.

To determine the Mn content in-situ, laser ablation–inductively coupled plasma–mass spectrometry (LA-ICP-MS) analysis was carried out on one polished slab of 11 of the studied specimens (Table 1). Equally spaced LA spots (midpoints: 85 μm apart; 65 μm diameter) were placed along the axis of maximum growth in the hinge plates. Analyses were performed with an Agilent 7500ce quadrupole ICP-MS coupled to an ESI NWR193 ArF excimer laser ablation system equipped with a TwoVol2 ablation cell. Pulse rate was set to 10 Hz at an energy density of 3 J/cm^2^. For each measurement, pre-ablation was set to 15 seconds followed by 25 seconds ablation time and 10 seconds wash-out time. NIST SRM 610 and 612 were used as calibration material [55] and accuracy and precision of the analyses were assessed by measuring the quality control materials (QCMs) USGS MACS-3, JCt-1 and USGS BCR-2G. The raw data were processed using the “LAtools” module [56] for python. For data reduction, ^43^Ca was used as the internal standard, applying the preferred values reported in the GeoReM database (http://georem.mpch-mainz.gwdg.de/, application version 27; [55, 57]) for the QCMs and calibration material. Element concentrations determined for the QCMs are given in S1 Dataset. In all quality control materials, the average relative Mn/Ca precision (standard deviation / mean) based on repeated measurements (n = 25) was better than 3.2%. Note that no measurements could be performed on the specimens cultured at 15°C (Table 1), because the hinge plates were contaminated by epoxy, which penetrated small cracks during preparation.

In addition to the microstructural analysis (see below) and the manganese method, growth patterns, i.e., growth increments and growth lines (periodic growth lines and disturbance lines) were studied in order to distinguish shell portions that formed in the field and under controlled laboratory conditions (e.g., presence or absence of annual growth lines). For this purpose, the polished section that was used for LA-ICP-MS analysis was immersed in Mutvei’s solution [58] for 7 minutes at 38°C under constant stirring. After rinsing in deionized water and air-drying, stained specimens were photographed using a Canon EOS 600D DLSR camera mounted to a Leica Stemi 508 stereomicroscope with sectoral dark-field illumination.

### Scanning electron microscopy and automated image analysis

The remaining polished shell slab of each specimen was analyzed by means of SEM (Phenom Pro Desktop SEM, 3^rd^ generation, equipped with a LaBr_6_ source and backscatter electron detector). To assess the microstructural properties of the laboratory-grown shell portions of the hinge plates, images were taken near the axis of maximum growth and calibrated for brightness and contrast. In these images, BMU size, elongation (ratio between the longest and shortest BMU axes) and coverage and pore size, were determined. Since the bivalves added variable amounts of shell material at the different temperature regimes [40], the regions selected for automated morphometric analyses varied in area between 1,218 and 47,479 μm^2^ (Table 1). SEM imaging for pore morphometry was conducted on non-sputtered, polished shell portions at 5 keV and 1,550× magnification. Qualitative images of the pores were taken on a fractured hinge plate of a specimen that was not used in the quantitative analyses (# 109).

To properly identify individual BMUs, specimens were immersed in 10.5 vol% H_2_O_2_ solution for 20 min to (superficially) remove the inter-crystalline organic matrix leaving behind empty spaces between the microstructural entities [59]. Note, since hydrogen peroxide is slightly acidic, this treatment also slightly attacked the BMUs and thus produced a three-dimensional relief (S1 File) with shell portions more resistant against H_2_O_2_ standing out of the surface (cores of BMUs) and less resistant shell portions forming depressions (inter-crystalline organics, pores and rims of BMUs). Thereafter, the samples were rinsed with demineralized water, gently dried with compressed air and sputter-coated with a 5 nm-thick platinum layer. Stitched overviews were generated from individual photographs taken at 5 keV and 3,200× magnification. Morphometric analyses of BMU size and coverage were conducted in SEM images taken at 10 keV and 7,700× magnification, which represented a compromise between sufficient resolution to discern individual BMUs and extensive image processing times.

**Fig 2:**
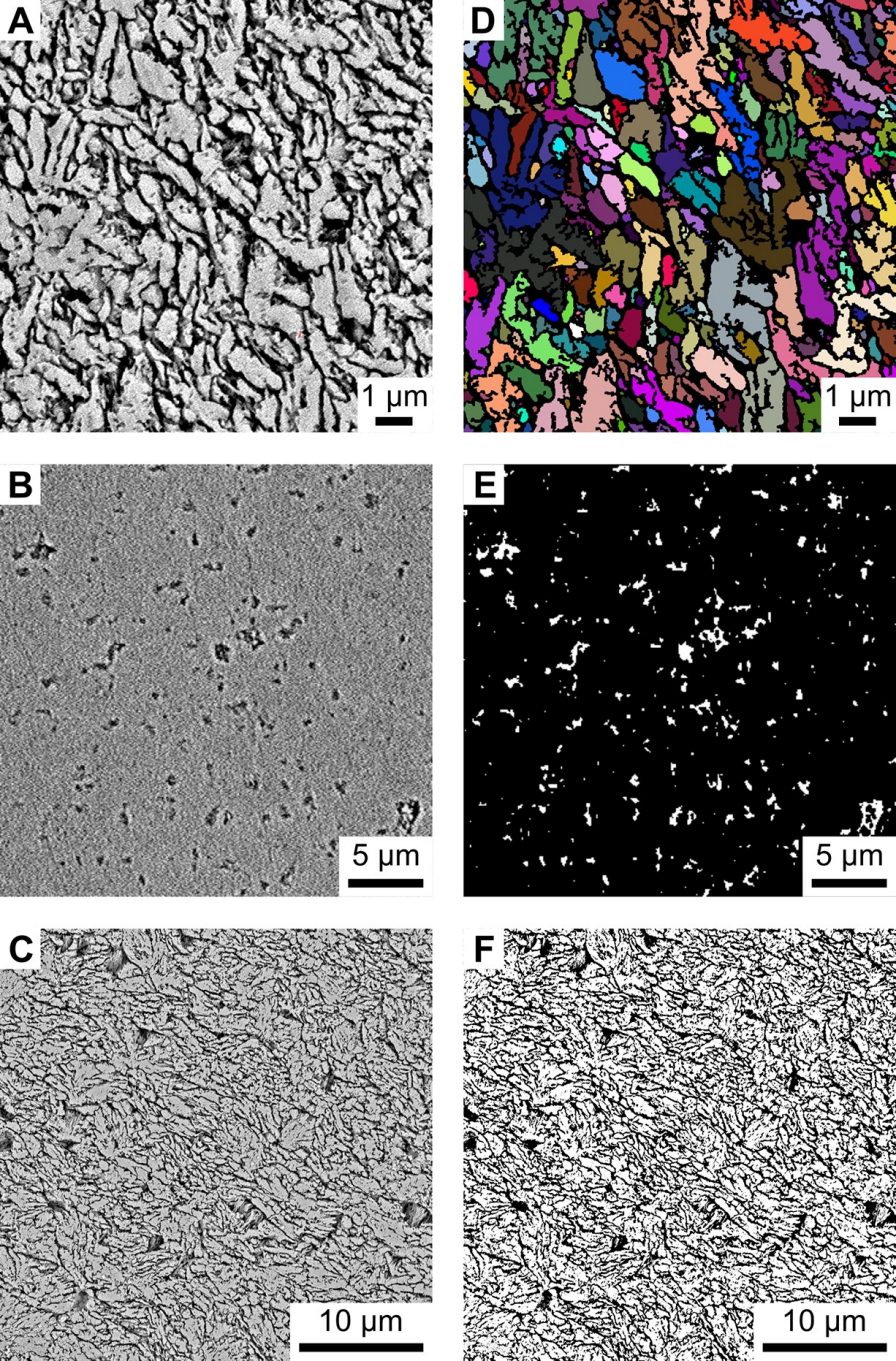
Image segmentation of hinge plate SEM backscatter images. (A-C) Morphological analyses were conducted on SEM backscatter images. BMU size and coverage were analyzed in the same image (A,C), taken after oxidation of the organic matrix by immersion in 10.5 vol% H_2_O_2_ for 20 minutes (image A magnified for visual clarity). Images for pore morphometry (B), in contrast, were taken in a polished and chemically untreated shell slab. (D-F) Binary images (objects of interest = white; remainder = black) used for the automated image segmentation process. Individual BMUs (D; here shown in various colors to allow discrimination of the individual entities) and pores (E, white) were recognized by the machine learning–based image segmentation process and separated from the remainder of the images (black). (F) For the calculation of BMU coverage a threshold based on the average gray value of the image series was applied. Values above this threshold were assigned to the crystalline (white) phase, those below the threshold to the inter-crystalline phase (black).

The BMU coverage-approach is based on intensity differences of the SEM backscatter images, which result from both the sample topography and the sample material. Removal of the inter-crystalline organic matrix by H_2_O_2_ oxidation eliminated material contrast and left behind a topographical gradient (visualized in SEM images as shadings of gray) between the BMUs (highest points) and the inter-crystalline space (lowest points). The mean intensity (i.e., mean gray value) of the images was set as a threshold value to better distinguish between the mineral phase (white) and inter-crystalline space (black) and generate black-and-white images (binarization; Fig 2B and 2D). The relative amount of white pixels in an image times 100 represents the (minimum) area in percent covered by crystalline phases (BMUs). It should be noted that the actual area occupied by the BMUs is larger than calculated here, because of the sample topography and the selected threshold value of the gray scale spectrum. Thus, black pixels represent inter-crystalline space as well as a proportion of BMU edges (Fig B in S1 File).

The automated measurement of the size (= area) of BMUs and pores as well as the elongation of BMUs required binary images, in which individual objects of interest were discretized and distinguished from the surrounding material. Recognition of these two phases (black = inter-crystalline spaces, pores and/or BMU rims; white = BMUs) was accomplished via machine learning–assisted image segmentation using the computer program Ilastik [60]. Performance of the machine learning method was evaluated by comparing the segmentation results to manually generated ‘ground truth’ segmentations (S3 File). Subsequently, the area of pores and BMUs as well as the elongation were automatically measured in the segmented binary images using the image processing software ImageJ [61–63]. Where necessary, artifacts (e.g., fractures in the shell, dirt particles and scratches on the sample surface) were manually excluded from morphometric analyses and only particles larger than two pixels were used for analysis.

### Machine learning–based image segmentation

In order to perform automatized recognition of BMUs and pores in the SEM images of etched and chemically untreated shell portions, respectively, we used the (bio)image analytical software Ilastik [60]. This program utilizes supervised machine learning [64] to sort image portions into certain categories (i.e., image segmentation) based on user-supplied training data. Ilastik was successfully applied in two- and three-dimensional pore space reconstructions of sedimentary rocks [65, 66]. In the present study, we employed the toolkit to classify each pixel of the SEM images by backscatter electron categories, whereby bright pixels reflected high-density mineral phases and black pixels empty spaces. In other words, the gray values of the SEM images were used as height indicators of the studied shell surfaces. The Ilastik workflow was supplied with training images (Fig 2A and 2C) and user-defined ‘labels’ (n ≥ 10 per image), with areas that belong to the class of interest (e.g., BMU, pore). The machine learning–based classifier also considers the image texture, gradients and edges [60] and thus has the potential to outperform solely intensity-based approaches. It produced binary images, where objects of interest (mineral phases and pores) were assigned the value 1 (white) and the remainder 0 (black; Fig 2D and 2E; BMUs shown in various colors instead of white to allow discrimination of the individual entities).

## Results

### Naturally and laboratory-grown shell portions

**Fig 3:**
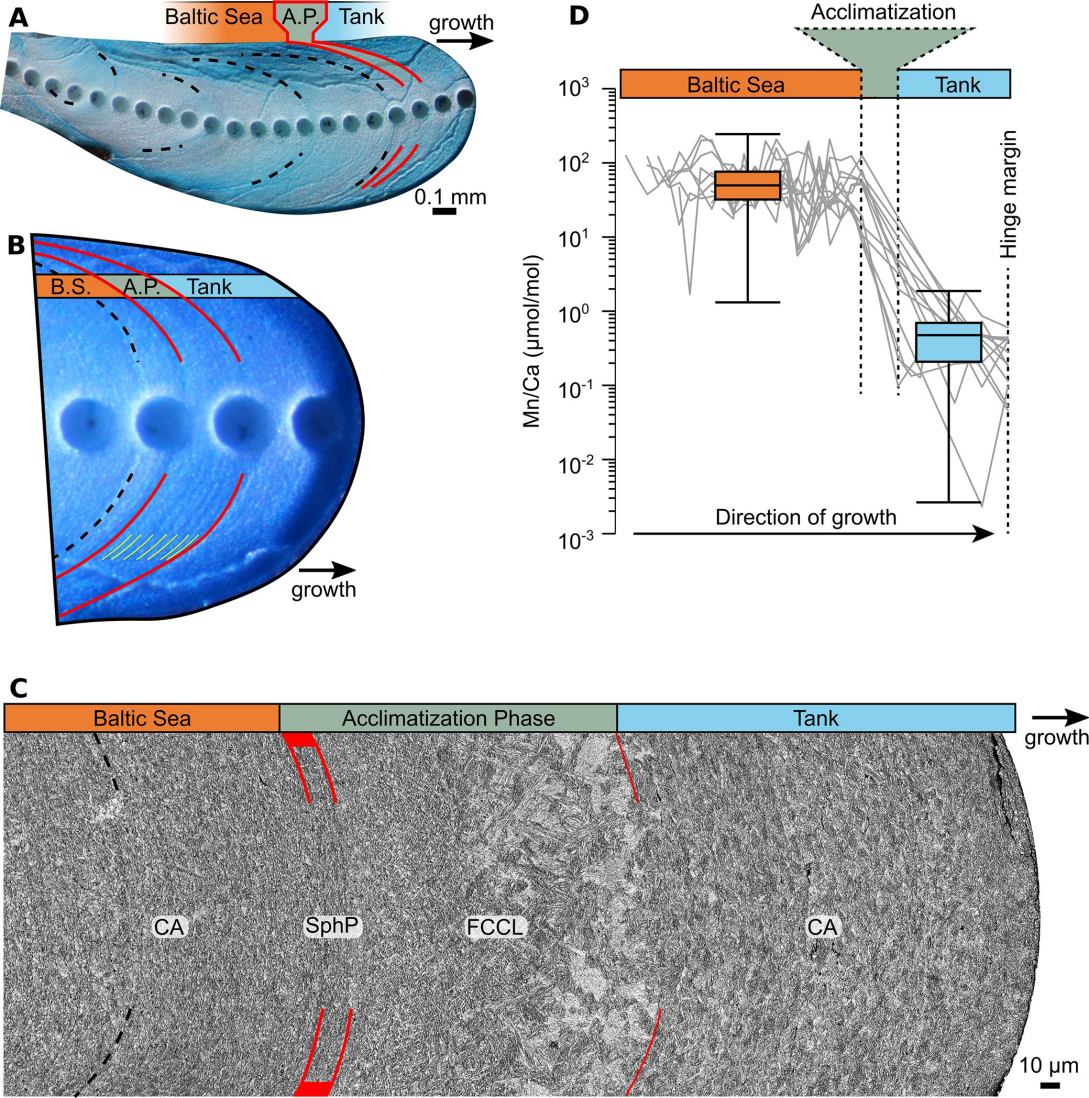
Growth patterns, Mn/Ca values and microstructures in the hinge plate of the studied *Arctica islandica*. (A) Mutvei-stained hinge plate of specimen Nioz-TC-12-A2R when viewed under a stereomicroscope with sectoral dark-field illumination. LA-ICPMS analysis was conducted prior to the immersion in Mutvei’s solution. Black dashed lines denote annual growth lines. Red solid lines = disturbance lines. These lines reflect handling stress (collection in the Baltic Sea, transferal to NIOZ, transferal to experimental tanks) and delimit the growth band that formed during the 4-week acclimatization phase. (B+C) Magnifications of (A): light microscopy (B) and SEM (C). (B) Portions formed during the acclimatization phase showed faint microgrowth patterns (the most defined ones indicated in yellow) possibly corresponding to circatidal or circalunidian increments and lines. Portion formed in tank was devoid of annual or disturbance lines. (C) Shell microstructures. Shell portions formed in the Baltic Sea consisted predominantly of crossed-acicular (CA) microstructure and exhibited variable sizes of the BMUs. The beginning of the experimental period is marked by a prominent spherulitic prismatic (SphP) disturbance line (red) followed by a zone of fine complex crossed-lamellar (FCCL) microstructure during the acclimatization phase. After another disturbance line, uniform CA microstructure is visible which formed in the experimental aquaria. (D) Ontogenetic changes of shell Mn/Ca ratios. Gray lines denote the different studied specimens; boxplots show distribution of manganese in shell portions formed in nature and during artificial tank environments.

**Fig 4:**
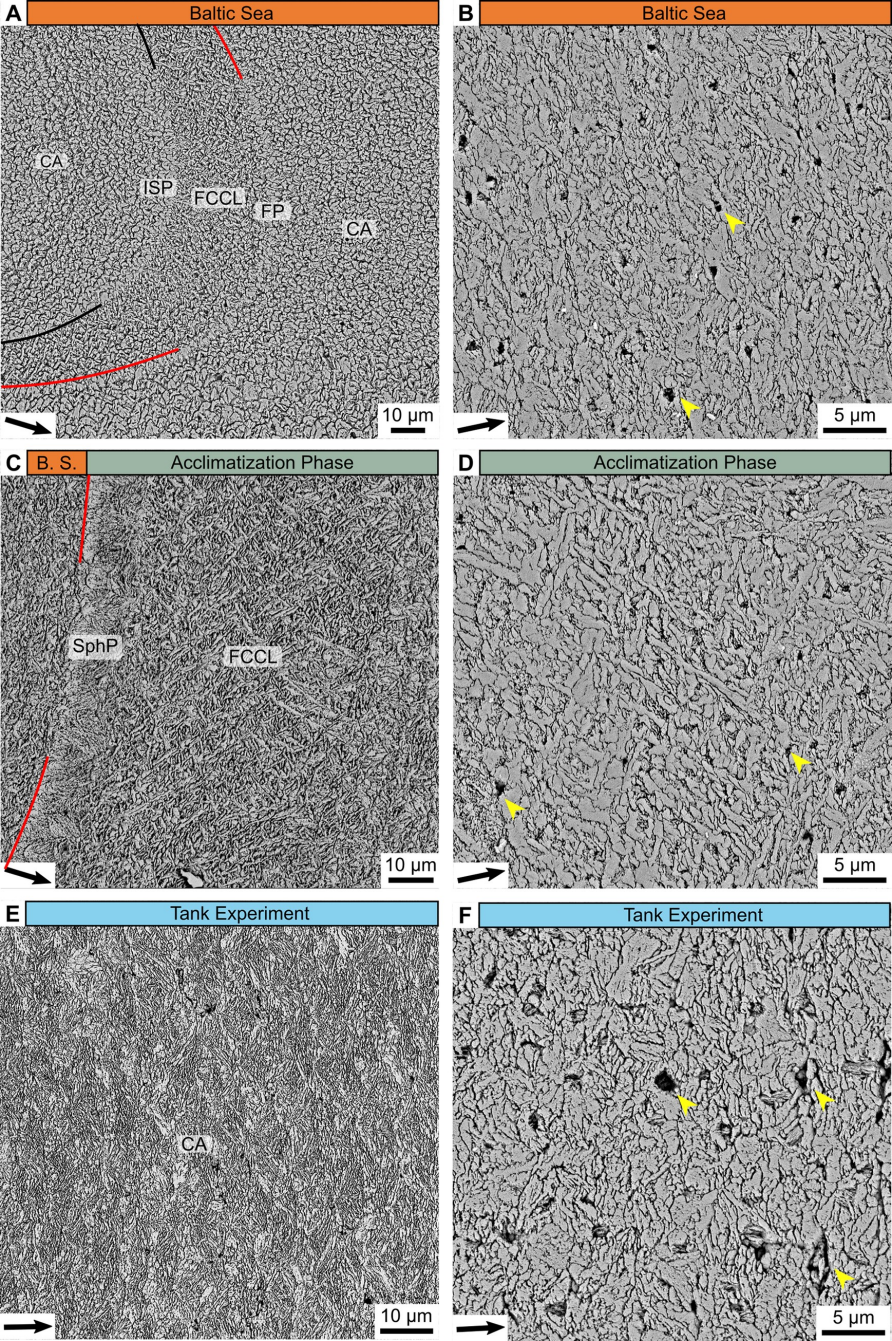
Microstructures in the hinge plate of the studied *Arctica islandica* specimens. (A) Hinge portions formed in the Baltic Sea predominantly exhibited crossed-acicular (CA) microstructure. Annual growth lines (black line) consisted of irregular simple prisms (ISP), often followed by 10–20 μm-broad fine complex crossed-lamellar (FCCL) fringes, as shown here. Numerous faintly visible disturbance lines (red line) consisted of fibrous prismatic (FP) or spherulitic prismatic (SphP) microstructures. (B) Magnified image of the CA microstructures formed in the Baltic Sea. BMUs consist of a few elongated crystallites, which merged with each other and were loosely aligned in two predominant directions (open angles in direction of growth). (C) Microstructures of shell portions formed in the Baltic Sea (B.S.; leftmost image portion) and the early acclimatization phase (rightmost image portion). A prominent disturbance line (red) composed of SphP microstructure delimits the two shell portions. During the acclimatization phase, FCCL microstructures were produced. (D) Magnified image of the FCCL microstructures formed during the acclimatization phase. Compared to CA microstructures, the FCCL BMUs are even more elongated, strictly aligned in two dip directions and intersect with their neighboring units. (E-F) The CA microstructures formed under controlled temperature in the laboratory exhibited a rather uniform appearance along the growth axis. Dip directions of the needle shaped BMUs varied more strongly than those formed in the Baltic Sea, leading to a looser arrangement and a less organized appearance. All microstructures–except ISP–contained nm- to μm-sized pores (yellow arrows). Black arrows indicate direction of growth.

All samples showed a prominent growth band (ca. 84.5 ± 32.2 μm broad) that divided the hinge plates into two zones which formed under natural and laboratory conditions, respectively. This growth band was also visible on external shell surfaces after the specimens were transferred to controlled-temperature tanks, but had not developed previously when collected in the field, confirming the assumption that the band formed during the one-month acclimatization phase. The two zones that were separated by this ‘acclimatization growth band’ were characterized by unique shell growth patterns and microstructural features (Fig 3A–3C). The ontogenetically younger zone before the acclimatization band, which formed in the Baltic Sea, occupied the largest proportion of the studied hinge plates (Fig 3A). In all studied specimens, this shell portion revealed four annual growth increments (predominantly CA microstructure; Fig 3C) which were delimited by distinct annual growth lines (ISP) with strong backscatter intensity and thus bright appearance (Fig 4A). The annual lines were in most cases followed by ca. 10–20 μm broad FCCL fringes composed of small acute crystallites that gradually increased in size along the growth direction (Fig 4B). We also observed several, erratically distributed, fainter growth lines, aka disturbance lines (reflecting physiologically stressful conditions [67–69], consisting of spherical prismatic (SphP) and fibrous prismatic microstructures (Fig 4A). These growth lines thus differ microstructurally from periodic growth lines (made of ISP), such as annual growth lines [28, 31].

The acclimatization band was developed shortly after the most recent annual growth line (Fig 3A and 3B). In Mutvei-stained specimens, it showed an internal color gradient from dark to light blue in the direction of growth (Fig 3A). At higher magnification, two–potentially fortnightly–growth increments became apparent within this growth band which were further subdivided into even narrower, up to 6.5 μm broad microgrowth increments (Fig 3B), likely resembling daily or circalunidian (lunar daily) growth increments. Considering that the bivalves were supplied with North Sea water during the acclimatization phase, the presence of tide-controlled growth patterns was expected.

**Fig 5:**
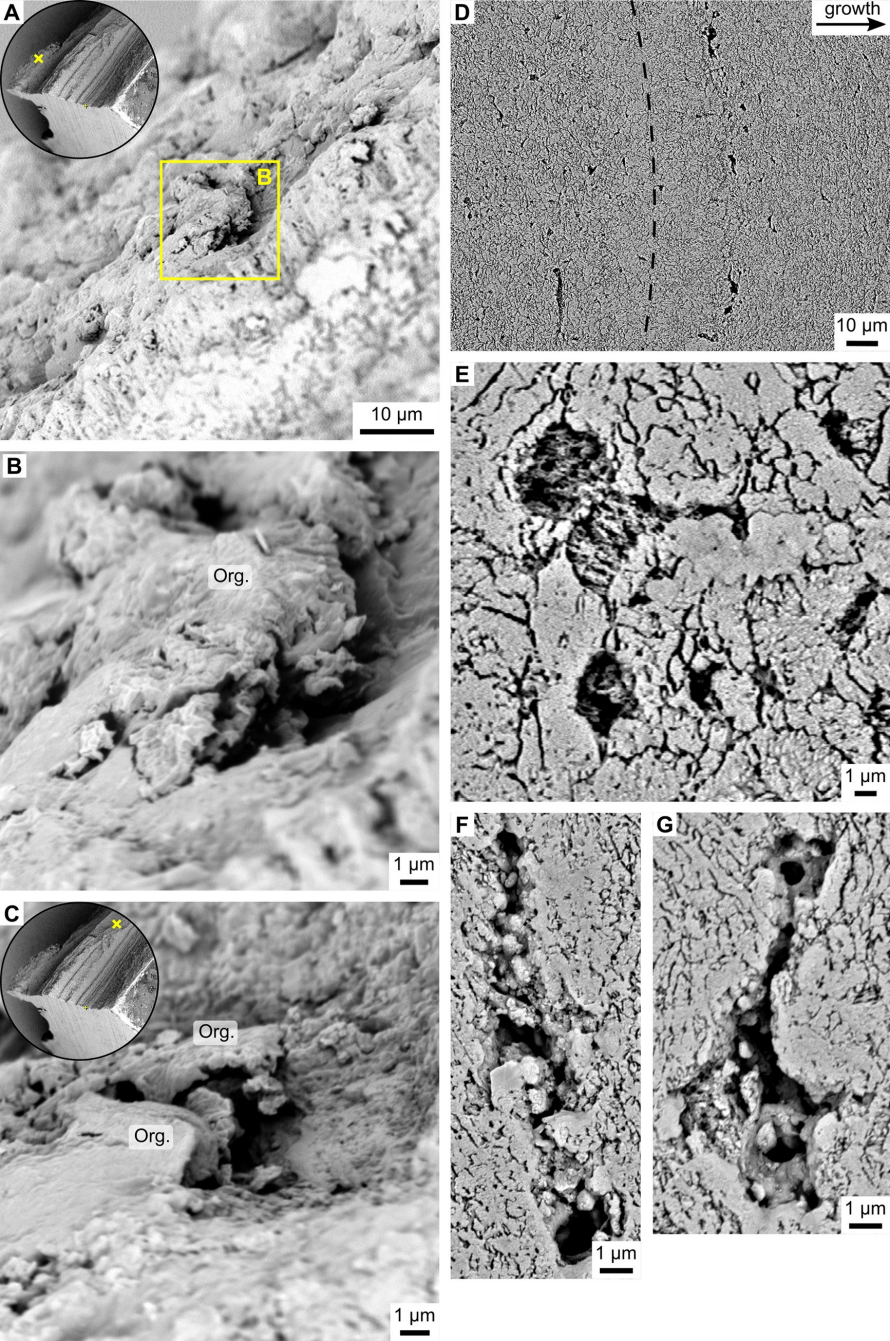
Pores of *Arctica islandica* shells. (A-C) Pores viewed on an untreated, fractured surface of a hinge plate. Insets in (A) and (C) depict an overview of the fractured shell. Yellow crosses within the insets denote the location of the respective images. (B,C) Higher magnification reveals the presence of shriveled organic envelopes (Org.) surrounding the partially void pores. (D-G) Pores viewed in a polished hinge surface after oxidation of the organic matter by immersion in 10.5% H_2_O_2_ for 20 minutes. Direction of growth is to the right. (D) Pores were aligned with their longest axis parallel to the growth front, as shown here near an annual growth line (black dashed line). (E) A pore filled with co-aligned, fibrous particles facing the same direction. (F,G) Pores partially filled with granular, spherical particles and remnants of organics.

Under the SEM, the acclimatization band appeared as a protruding ridge composed of FCCL microstructure which was delimited by a disturbance line made of SphP microstructure. The aforementioned color change in Mutvei-stained sections was associated with a gradually rising gray value (i.e., increasing backscatter electron intensity, brighter) in the SEM images resulting from increasingly larger FCCL BMUs that progressively merged into triangular patches (Fig 3C). Overall, the acclimatization band consisted of very thin, highly elongated, needle shaped BMUs that were aligned in two predominant dip directions (open angle in the direction of growth; Fig 4C and 4D). In growth direction, the acclimatization band ended with another disturbance line followed by a shell portion (representing the 95 days under controlled temperature conditions) composed of CA microstructure. The CA microstructure precipitated during the experimental phase differed from that in ontogenetically younger portions preceding the acclimatization band by more equally sized and rounder BMUs (Fig 4E and 4F). This more uniform CA microstructure dominated the remainder of shell up to the margin of the hinge plate, i.e., the date of death of the animals (Fig 3A–3C). Individual BMUs of the CA microstructure typically consisted of (two to four) fused needles (Fig 4F). Like the naturally formed shell portion, the laboratory-grown zone contained a considerable number of μm-sized pores that were often filled with granular (Fig 5E) and/or fibrous (Fig 5F and 5G) crystallites. Fractured shell portions demonstrated that the pores were originally coated by and infilled with organic material (Fig 5A–5C).

The change in microstructure in the direction of growth was accompanied by a marked change in shell Mn/Ca values. Within the acclimatization band, Mn/Ca ratios abruptly decreased in all specimens, from 502.39 ± 377.24 μmol mol^-1^ to merely 4.26 ± 2.40 μmol mol^-1^ (median ± 1 inner quartile range; Fig 3D, S1 Dataset). Mn/Ca provided additional evidence for which shell portions formed in the Baltic Sea and during the 95 days spent under temperature-controlled conditions in laboratory tanks.

### Microstructure morphometrics of shell portions grown at constant temperature regimes

**Fig 6:**
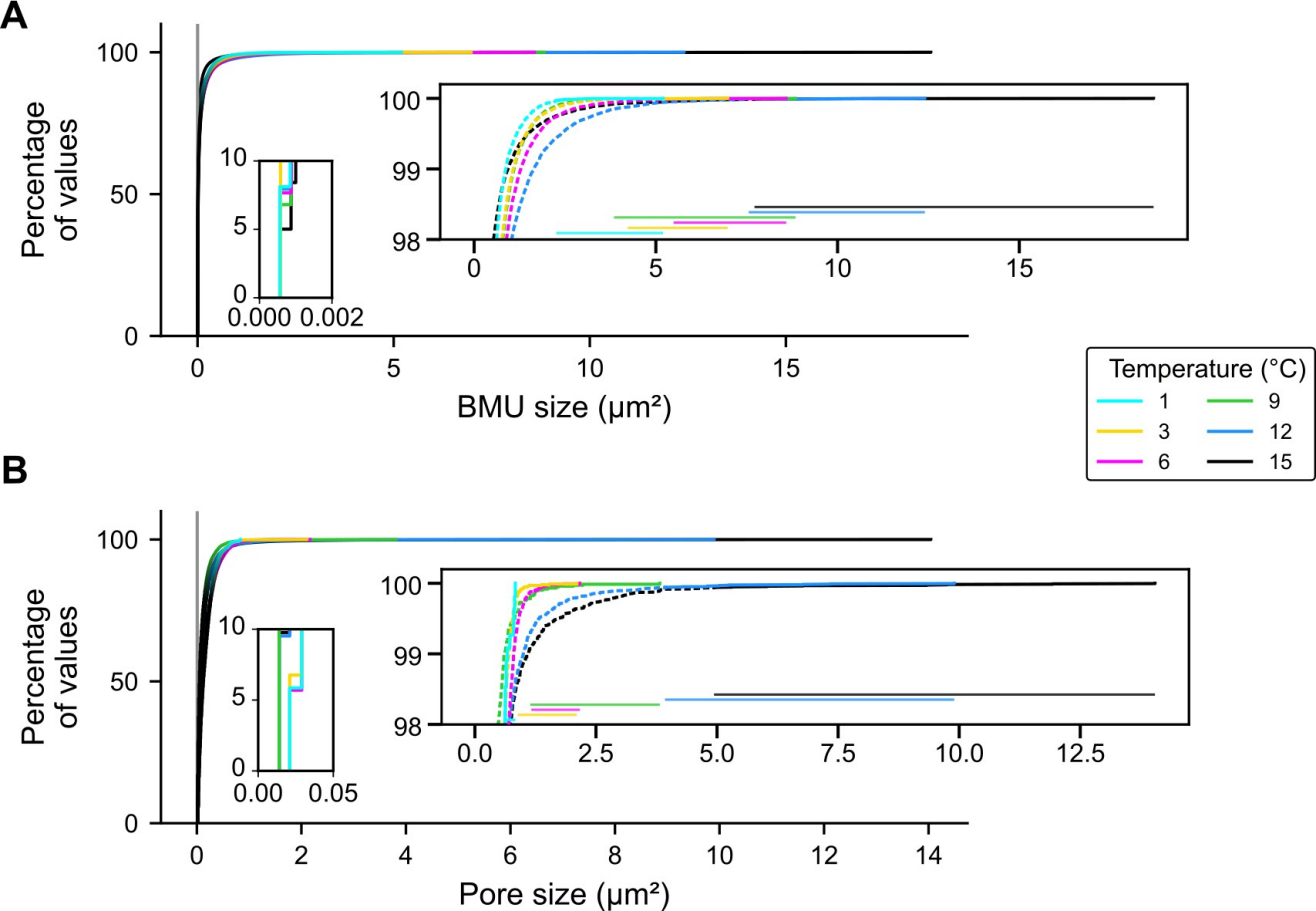
Empirical cumulative distribution functions of (A) BMU size and (B) pore size. The majority of BMUs and pores incorporated in the shells were small, whereas only few large entities existed. Small insets depict magnifications of the smallest 1% of the values and the large insets depict the largest 2% of the values (dashed lines). Ranges of the 15 largest values (solid lines) are shown as horizontal lines. The maximum size of pores and BMUs increased with culturing temperature. Detection limit of the analytical method (2 pixels) is indicated represented by vertical gray line.

**Fig 7:**
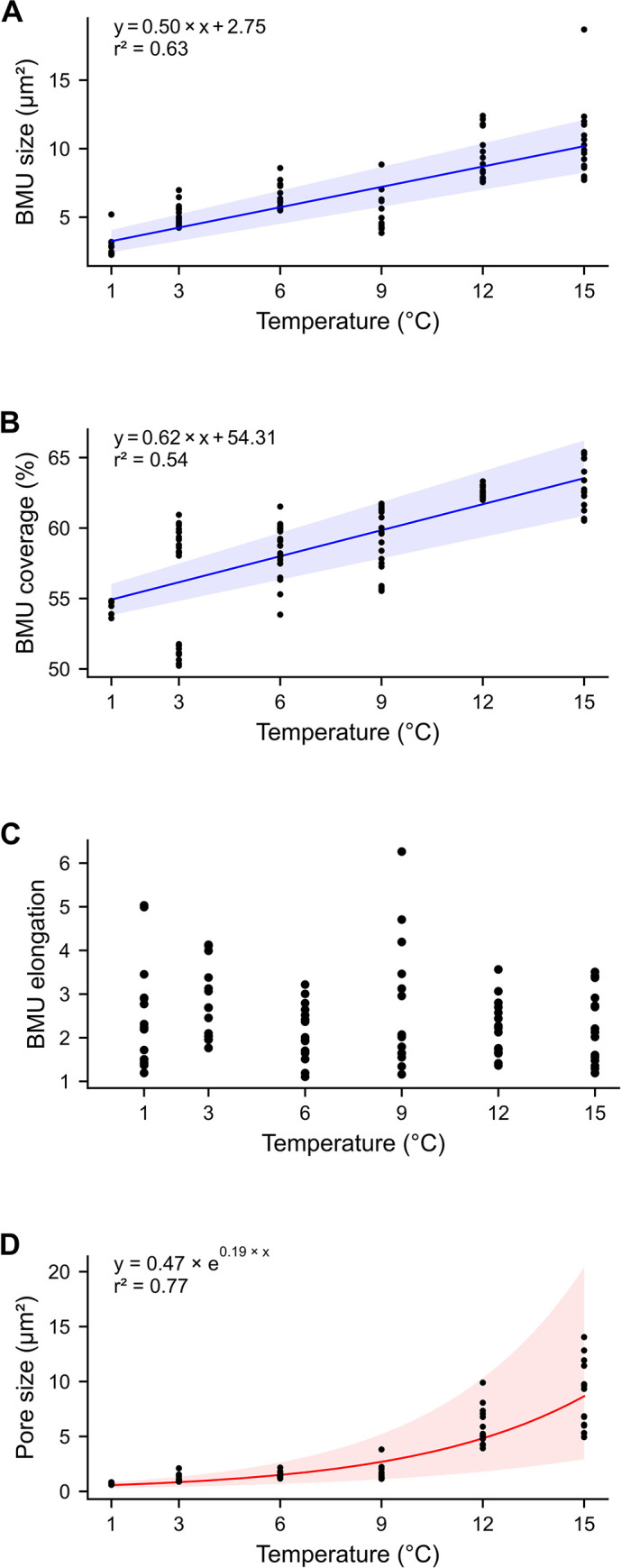
Quantitative microstructural data of hinge plate grown under temperature-controlled conditions. (A) The size of the 15 largest BMUs of each temperature treatment and (B) the space occupied by crystalline phase (BMU coverage) displayed a statistically significant linear increase with culturing temperature. Note, the relationship between BMU size and temperature was stronger than that of the BMU coverage values (r^2^ = 0.67 vs. 0.54). Large inter-individual variability was observed among the BMU coverage values of specimen cultured at 3°C. (C) BMU elongation did not exhibit any significant link with culturing temperature. (D) The size of the largest 15 pores increased exponentially with rising temperature. Shaded areas represent ± 2 standard errors of each parameter of the respective models. Model equations and r^2^ goodness-of-fit values are annotated in the graphs. For justification of the choice of sample size (15 largest values) and type of model see S2 File.

BMU and pore sizes exhibited logarithmic distributions with a distinct predominance of very small entities (99.9% of values fell below 3.13 and 2.79 μm^2^, respectively; Fig 6). The size of the BMUs ranged from 0.0005 to 18.70 μm^2^ (Fig 6A), while that of the pores varied between 0.003 and 14.04 μm^2^ (Fig 6B, S2 Dataset). Distribution curves of BMU and pore sizes revealed statistically significant differences between culturing temperatures in all cases, except for pore sizes between 1 and 3°C (two sample Kolmogorov-Smirnov tests, *p* < 0.05; Fig 6, S2 Dataset). Thereby, the largest BMU and pore size values increased most strongly with culturing temperature, whereas the large amount of smaller BMUs and pores remained largely invariant. In other words, when considering increasingly large data subsets of the largest pores and BMUs, the correlation to water temperature decreases (S2 File). To balance between explanatory power and sufficient sample size, a subset of the 15 largest BMUs and pores of each temperature setting was considered for the construction of the predictive models. These data subsets revealed statistically significant positive correlations to culturing temperature (Fig 7, S2 Dataset). The size of the 15 largest BMUs increased from 2.79 ± 0.74 μm^2^ at 1°C to 10.42 ± 2.74 μm^2^ at 15°C (Spearman’s r = 0.82, r^2^ = 0.67, *p* < 0.05; Fig 7A). Linear and exponential models between BMU size and temperature exhibited a near complete overlap. Therefore, linear models were chosen (Fig B in S2 File). The size of the 15 largest pores, in contrast, increased exponentially from 0.68 ± 0.09 μm^2^ at 1°C to 10.13 ± 2.41 μm^2^ at 15°C (r = 0.92, r^2^ = 0.77, *p* < 0.05; Fig 7D). Here, linear models produced negative values and non-randomly distributed residuals. These problems did not occur when exponential regressions were computed (see Fig B in S2 File). Although BMU coverage did not require thresholding, respective data showed the same trend as BMU size, i.e., a statistically significant positive linear coupling with water temperature (r = 0.78, r^2^ = 0.54, *p* < 0.05; Fig 7B). The area covered by BMUs increased linearly from 55.4 ± 0.5% at 1°C to 63.0 ± 1.7% at 15°C. No correlation was found between the elongation of the 15 largest BMU and temperature (Fig 7C).

## Discussion

### Water temperature control on microstructural properties

As revealed by the findings of this study, several microstructural properties in the hinge plate of laboratory grown *Arctica islandica* specimens were statistically significantly linked to water temperature. Specifically, the size of the largest BMUs and pores as well as the relative proportion of the shell occupied by biominerals increased with temperature (Fig 7). The correlation between BMU size and temperature was also highlighted in other existing studies on biominerals of mollusks [16, 24–26] pointing to an overarching control mechanism. Such a concept was recently outlined by Milano et al. [25] and will be briefly rehearsed in the following. As in abiogenic systems [e.g., 70], rising water temperature can promote the amount and rate of CaCO_3_ precipitation. This requires sufficient availability of calcium and bicarbonate ions. In bivalves, these building materials are transported from seawater across epithelial membranes to the site of biomineralization, for example, via passive diffusion through ion channels and actively via ion pumps [71, 72]. Both transport mechanisms proceed faster at higher temperature [71, 72]. If species-specific optimum growth temperatures are exceeded, the rate of transmembrane ion transport via ATPase Ca^2+^-pumps [71] decreases and can potentially result in reduced biomineralization rates.

However, this model does not seem to be directly applicable to the CA microstructural entities in *A*. *islandica* shells. Actually, the overwhelming majority of BMUs and pores seemed to remain largely unaffected by the prevailing temperature regime (Fig 6). One possible explanation for this surprising observation is the morphology of the BMUs. The majority of angles at which the long, needle shaped (acicular) objects of CA microstructure can be sectioned produce cutting patterns with small areas. This cut-effect bias explains the shape of the size distribution curve in Fig 6. Significant correlation existed also between the size of (more elongated) BMUs which were cut parallel to the longest axes, but fell below the selected threshold (15 largest BMUs of each temperature regime; Fig A in S2 File). Respective cutting planes may be identified through a study of the crystallographic orientation, since EBSD (Electron Backscatter Diffraction Analysis) results demonstrated that the a- and b-axes of the BMUs can assume multiple orientations [33] and do not always coincide with the same morphological axis Future studies should implement EBSD analyses.

The reservation must be made that the elongation of the 15 largest BMUs of each temperature setting was uncoupled from temperature, i.e., in the studied specimens of *A*. *islandica*, higher temperature did not favor the growth of more elongated CA needles. It is therefore likely that the same applies to the remaining BMUs and elongation is not tied to temperature. Since this observation contradicts previous observations by Milano et al. [25] on BMUs in the nondenticular composite prismatic microstructure of naturally-grown *Cerastoderma edule*, it is hypothesized here that the shape of BMUs, at least the largest ones, is more strongly affected by other environmental variables which were successfully precluded in controlled laboratory conditions. Perhaps, the temperature control on BMU shape is also species-specific and/or microstructure-specific. More detailed investigation of this matter would certainly be useful.

An alternative, more likely explanation for the relationship between temperature and microstructural properties in studied shell portions of *A*. *islandica* is that the growth of most BMUs was strongly biologically limited, whereas only a few entities were allowed to grow larger. These few BMUs were governed to a larger degree by thermodynamic processes and thus carried a stronger temperature signal than the smaller ones. Perhaps, a clear dominance of small BMUs was required for biomechanical reasons, because the hardness of shell material decreases with increasing BMU size [73]. Interestingly, the size of the smallest BMUs (represented by the lower end of the size distribution curve; magnified portion in Fig 6) remained close to the detection limit of two pixels, irrespective of different growth temperatures. This either suggests that the number of BMUs in a given area of the growth front remained unchanged, or that noise in the SEM backscatter images was erroneously detected as tiny BMUs. In case of the latter, the effect could be counteracted by using longer pixel integration times during SEM imagery, or, as in our case, by applying an analysis threshold (S1 File).

A strong biological control also appears to have limited the size of pores. As in the case of BMUs, the relationship between pore size and water temperature was most pronounced when only a few of the largest pores were considered (Fig 7D, Fig A in S2 File). In order to interpret why temperature dependence amplifies at larger pore sizes, more investigation is needed on the function of the pores, which as yet remains unresolved [28, 30, 31]. The exponential nature of the temperature relationship of pore size (Fig B in S2 File) possibly suggests a strong influence of metabolic processes, because these also accelerate exponentially in warmer temperatures [74]. Furthermore, the orientation of the pores’ long axes along the growth front (Fig 5D) might indicate an interrelation with biomineralization processes. In accordance with previous studies [33], the pores were coated and filled with organic components and contained mineral precipitates. Other than the tubules in, e.g., arcoid bivalves [75–77], the pores in *A*. *islandica* shells were only a few micrometers in size, irregular in shape and often elongated, but spatially highly confined. Although many pores were cross cut at arbitrary angles, none was observed that showed a tube-like morphology. Hence, the pores are not tubular channels (as stated in [30, 39]), precluding a sensory function.

It appears unlikely that the pores represent dump sites for organic matrices that were produced in excess of demand during biomineralization, because the production of organics is very energy-consumptive [78]. However, the pores could represent the remains of failed attempts to form BMUs. This hypothesis would take up the ACC-mediated biomineralization hypothesis according to which organic envelopes (compartments) are produced, which are subsequently filled with amorphous carbonate that later transforms into a crystalline phase [79–81]. However, this type of biomineralization occurs in mollusks with nacro-prismatic shells [81, 82] with high organic content (ca. 3.5 wt%, [83]) and likely does not play a major role in *A*. *islandica* shells with much lower amounts of organics (1.65 wt%, [83]). Furthermore, nacro-prismatic shells contain sheets of β-chitin and silk-like proteins [84], which are required for the building of such organic envelopes. Shell organics of *A*. *islandica*, on the contrary, contain less of these compartment-forming molecules, but more polar and soluble organic components, which closely resemble the composition of the organic matter in the crossed-lamellar microstructure [83, 85]. Actually, the nucleation of crossed microstructures has been shown to be largely unaffected by organic scaffolding [79]. Presumably, organic compounds of *A*. *islandica* shells predominantly provide the nucleation sites for the shell carbonate, control the biomineral precipitation rate, and the trace metal composition. In conclusion, the function of pores in *A*. *islandica* remains enigmatic, but a role in biomineralization appears likely. As such their sizes are largely governed by biological processes and partly by thermodynamic processes.

### Data quality and method evaluation

Given the novel analytical approaches used in the current study to assess the link between temperature and microstructural properties, an appraisal of the working principles and image processing techniques seems justified.

At the outset we wish to emphasize the importance of conducting controlled laboratory experiments when it comes to assess new proxies for environmental variables. Only under such conditions it is possible to study the link between a specific environmental variable and microstructural properties because growth conditions can be selectively modified. On the other hand, an artificial growth environment may evoke the formation of untypical microstructural patterns [86]. As demonstrated here, shell portions in the hinge plate formed under laboratory conditions deviated from those grown in nature. The CA microstructure of the laboratory-grown shell portions appeared more uniform with rounder and more equally sized BMUs than in the shell portion that formed in the Baltic Sea (Fig 4). Therefore, the results achieved herein need to be tested in the future using field-grown specimens. Obviously, this requires detailed and high-resolution monitoring of a variety of environmental properties, which were not available in the present study for the shell portions produced in the Baltic Sea.

In this study, the discrimination and recognition of microstructural entities was based on gentle variations in surface topography and as such intensity variations caused by electrons backscattered by the shells. Because of the fixed position of the backscatter electron detectors, such variations depend on the orientation of the studied surface in the SEM. The largest number of backscatter electrons is detected when the sample surface is in horizontal position, and values progressively decrease with increasing sample tilt [87]. Accordingly, backscatter intensity in a mineralogically uniform material, e.g., an aragonitic shell, can be used to trace changes in surface height. Thereby, polished surfaces of individual BMUs form elevations that emit high backscatter intensity (high gray values, light gray to white), whereas depressions (such as the pores or the inter-crystalline space; here produced by immersion in H_2_O_2_) are detected as areas of reduced to zero emission (low gray values, dark gray to black) (Fig 2, S1 File). Such topographic principles are well understood [88] and have been employed in several existing studies to assess the microstructural properties of bivalve [26] and gastropod shells [89] as well as other calcium carbonate materials [90].

A reliable automated image segmentation process represents a fundamental step to identify individual BMUs and pores and subsequently conduct morphometric analyses. Because of the large gray value contrast between the polished shell surface and the pores, segmentation of these microstructural entities did not require intensive image pretreatment or training of the (bio)image software Ilastik. The precise identification of pore boundaries was occasionally complicated by the presence of particles inside the pores (Figs 2 and 5E–5G). However, when supplied with additional training data to segment these structures, the classification algorithm overcame such bias and produced reliable pore space reconstructions (Fig 2). The automated segmentation process of the pores allowed use of a lower magnification (1,550×) than required for the BMUs, which considerably reduced the acquisition and processing time while maintaining an adequate spatial resolution.

On the contrary, training of the software for BMU recognition turned out more challenging. A major prerequisite to use automatized image segmentation is that all images have uniform characteristics, e.g., comparable surface topography generated by etching (of the mineral phase) or oxidation (of the organic phases) and comparable image resolution. To assess the BMU size and coverage, the polished shell surfaces were immersed in H_2_O_2_ to reveal boundaries between the microstructural units. We decided to treat the sample with an oxidative agent to remove organic matrices located between the BMUs (inter-crystalline organics) without completely dissolving the mineral phase of the shell. In preparation for the present study, however, we experimented with different chemical treatments of *A*. *islandica* shells to identify the most suitable method. Among others, different types and concentrations of acids and oxidation agents were explored (Fig A in S1 File). According to these tests, immersion in formic or hydrochloric acid, even at very low concentration (< 0.01 vol%), resulted in micrometer-scale to nanometer-scale dissolution of the polished BMU surfaces. These areas were then erroneously detected by the segmentation software as BMU boundaries. Furthermore, the etching affected the individual BMUs differently, probably related to minute variations in density and/or intra-crystalline organic content. The degree of etching also varied considerably among specimens. A solution of 10.5 vol% H_2_O_2_ for 20 minutes turned out to provide the best results for the scope of the present study. Hydrogen peroxide solution has been used in the past to remove organic matter from biogenic carbonates [59, 91, 92]. It reacted well with the organic matrices of the shell, which consist mostly of non-acidic, polar proteinaceous materials [83]. According to high-resolution images, BMUs remained largely unaltered by treatment with H_2_O_2_ (Fig 2A), except for the rims (Fig B in S1 File), and consistent results were achieved within and among specimens. These assets were prerequisite for a reproducible segmentation process. Note, that the large inter-individual variability observed in the BMU coverage values of specimens cultured at 3°C (Fig 7) does likely not result from the chosen preparation and etching method, because those specimens were embedded in the same batch of epoxy and thus prepared in exactly the same manner.

Through the use of novel analytical techniques, we were able to automate the recognition of microstructural entities as well as the morphometric analyses, which hitherto had to be performed manually or semi-automatically with manual corrections [89, 93]. Previous morphometric studies of BMUs included only few SEM images representing relatively small shell areas (e.g., 19 images in [25], 64 images in [94]) in order to compensate for the excessively long analytical times. With such reduced sample sizes, the chances of finding relevant relationships between microstructural properties and environmental variables diminish, as shown by the extraordinarily high threshold that had to be chosen in the present study in conjunction with the logarithmic particle size distributions (Fig A in S2 File; Fig 6). The technique used here, however, reduced the time for measurement significantly and facilitated the analysis of large shell regions (Table 1) without sacrificing analytical precision or requiring manual intervention (S3 File). Accordingly, the sample size and spatial coverage (total number of BMUs = 699,587) substantially exceeded those of previous studies (e.g., ca. 2,170 BMUs in Milano et al., 2017a). These large sample sizes enabled us to detect subtle temperature-induced variability of the shell microstructural properties that previously remained unnoticed, emphasizing the importance of comprehensive shell imaging and highlighting the potential of automated image processing techniques combined with machine learning algorithms.

Focusing on a specific subset (thresholding) can serve as an efficient means to detect unnoticed signals in large datasets. This approach has been successfully applied in a previous study of the crossed-lamellar microstructure of *Glcymeris bimaculata* [26], in which only the largest 10% of the biominerals were taken into consideration. The appropriate threshold value most certainly varies among different microstructures due to differences in the degree of hierarchical organization, complexity, the morphology of individual BMUs and fusing of discrete BMUs to larger units. For example, in the crossed-lamellar microstructure, stacks of the smallest building blocks, elongated rods of a few hundred nm diameter, so-called 3^rd^ order lamellae, form 2^nd^ order lamellae, which are assembled into 1^st^ order lamellae. The latter can attain widths of ca. 28 μm and are alternatingly arranged in a rustic fence-like manner [89, 95–97]. The CA microstructure, in contrast, lacks such a hierarchical organization into larger structural units. Their BMUs consist of acicular units, which are only co-aligned morphologically in two dominant dip directions (ca. 30–40° off the growth surface) and fused together in platelets [95, 98]. EBSD experiments performed on shell hinges of *Arctica islandica* confirmed that the morphologically co-aligned acicular units also share a common crystallographic orientation (thus forming a BMU) [33]. The crystallographic c-axes of all BMUs are aligned roughly parallel to the direction of growth, but other than that, no overarching crystallographic alignment seems to persist [33]. In conclusion, the BMUs of the CA microstructure are much smaller and less complexly arranged than the BMUs of the crossed-lamellar microstructure. Cross-sections of the CA microstructure reveal more irregularly shaped BMUs than those of other microstructures (i.e., nacre tablets [24], prisms [25], 3^rd^ order lamellae of the cross-lamellar microstructure [26]). Therefore, a much higher size threshold was required than in previous analyses (S2 File). The same issue arose in the case of pores, which were likewise irregular in shape.

The BMU coverage analysis followed a more simplistic approach than the segmentation analysis and was based on the proportion of shell covered by crystalline phases relative to such largely occupied by organic phases plus downward dipping edges of BMUs. The gray value of each pixel of the binarized image indirectly provided height information over an area of ca. 0.0003 μm^2^ (= the same resolution as used for BMU size analysis), spanning a total topographical height of ca. 6 μm. The production of binary images depended strongly on the uniformity of the gray scale balance among all studied SEM images. The latter is a measure of the emitted backscatter electrons during acquisition and can strongly vary between specimens, depending on subtle differences in thickness of the platinum coating, orientation of the studied surface or density of the aragonite. Since the individual images used in this study exhibited similar gray value distributions, the average gray value of all studied images was chosen as the threshold to distinguish BMUs from the inter-crystalline space. In other words, the BMU coverage serves as a measure of surface area above the average height of all samples, inferred from backscatter intensities (gray values; Fig B in S1 File). In turn, this means that the BMU coverage value is no exact measure of the actual proportion occupied by BMUs (see section 2.3). The BMUs stand out from the surface as plateaus, but the angle at which their rims dip downward is shallower than 90°. Due to the selected gray value threshold, some portions of these gently descending BMU edges were assigned to the depression zones and became black in the binarized image. Hence the BMU coverage values cannot be used to quantify the actual proportion of inter-crystalline space or BMUs. However, it can be used to rapidly detect differences in the relative proportion of crystalline phase in different shell portions. BMU coverage results agreed with that obtained from segmented BMU analysis documenting the validity of the method.

## Conclusions

Variations in water temperature resulted in subtle microstructural changes in the hinge plate of *A*. *islandica* grown under controlled laboratory conditions. The size of the largest biomineral units and pores as well as the relative proportion of crystalline components in the crossed-acicular microstructure increased with water temperature. Since only a very small proportion of the microstructural units was affected, the subtle changes would have remained unnoticed without the use of automated image processing techniques and machine learning–assisted object recognition. While it may be challenging to reliably reconstruct small (1°C) changes of long-term ocean climate from these minute microstructural variations, they can prove useful to assess seasonal temperature variations and extremes or serve as a source of temperature information when geochemical proxy signals have vanished due to diagenetic overprint. Further using fossil shells collected from different localities and environmental regimes will shed light on the applicability and sensitivity of the new microstructure-based temperature proxies.

## Supporting information

S1 Dataset Mn/Ca data (LA-ICP-MS) of the studied bivalve shells. (XLSX)

S2 Dataset Morphological data of the BMUs and pores. (XLSX)

S1 File Sample preparation for morphometric analyses. Results of experiments to identify the suitable preparation method and three-dimensional model of the sample topography in SEM.
(DOCX)

S2 File Statistical analyses of morphometric data. Information on the selection of a suitable analysis threshold and justification for the choice of linear and exponential models, respectively.
(DOCX)

S3 File Evaluation of the image segmentation process. (DOCX)

## References

[1] Schmidt GA, Annan JD, Bartlein PJ, Cook BI, Guilyardi E, Hargreaves JC, et al. Using palaeo-climate comparisons to constrain future projections in CMIP5. Clim Past. 2014;10: 221–250. 10.5194/cp-10-221-2014

[2] Cauquoin A, Werner M, Lohmann G. Water isotopes—climate relationships for the mid-Holocene and preindustrial period simulated with an isotope-enabled version of MPI-ESM. Clim Past. 2019;15: 1913–1937. 10.5194/cp-15-1913-2019

[3] Asami R, Yoshimura N, Toriyabe H, Minei S, Shinjo R, Hongo C, et al. High-resolution evidence for Middle Holocene East Asian winter and summer monsoon variations: snapshots of fossil coral records. Geophys Res Lett. 2020;47: e2020GL088509. 10.1029/2020GL088509

[4] Butler PG, Wanamaker AD, Scourse JD, Richardson CA, Reynolds DJ. Variability of marine climate on the North Icelandic Shelf in a 1357-year proxy archive based on growth increments in the bivalve *Arctica islandica*. Palaeogeogr Palaeoclimatol Palaeoecol. 2013;373: 141–151. 10.1016/j.palaeo.2012.01.016

[5] Justine D, Gwénaëlle C, Pierre P, Pascal L, André P, Laurent C, et al. Assessment of Ba/Ca in *Arctica islandica* shells as a proxy for phytoplankton dynamics in the Northwestern Atlantic Ocean. Estuar Coast Shelf Sci. 2020;237: 106628. 10.1016/j.ecss.2020.106628

[6] Reynolds DJ, Butler PG, Williams SM, Scourse JD, Richardson CA, Wanamaker AD, et al. A multiproxy reconstruction of Hebridean (NW Scotland) spring sea surface temperatures between AD 1805 and 2010. Palaeogeogr Palaeoclimatol Palaeoecol. 2013;386: 275–285. 10.1016/j.palaeo.2013.05.029

[7] Featherstone AM, Butler PG, Schöne BR, Peharda M, Thébault J. A 45-year sub-annual reconstruction of seawater temperature in the Bay of Brest, France, using the shell oxygen isotope composition of the bivalve *Glycymeris glycymeris*. The Holocene. 2019; 1–10. 10.1177/0959683619865592

[8] Schöne BR, Freyre Castro AD, Fiebig J, Houk SD, Oschmann W, Kröncke I. Sea surface water temperatures over the period 1884–1983 reconstructed from oxygen isotope ratios of a bivalve mollusk shell (*Arctica islandica*, southern North Sea). Palaeogeogr Palaeoclimatol Palaeoecol. 2004;212: 215–232. 10.1016/j.palaeo.2004.05.024

[9] von Leesen G, Beierlein L, Scarponi D, Schöne BR, Brey T. A low seasonality scenario in the Mediterranean Sea during the Calabrian (Early Pleistocene) inferred from fossil *Arctica islandica* shells. Palaeogeogr Palaeoclimatol Palaeoecol. 2017;485: 706–714. 10.1016/j.palaeo.2017.07.027

[10] Casella LA, He S, Griesshaber E, Fernández-Díaz L, Greiner M, Harper EM, et al. Hydrothermal alteration of aragonitic biocarbonates: assessment of micro- and nanostructural dissolution–reprecipitation and constraints of diagenetic overprint from quantitative statistical grain-area analysis. Biogeosciences. 2018;15: 7451–7484. 10.5194/bg-15-7451-2018

[11] Eiler JM. Paleoclimate reconstruction using carbonate clumped isotope thermometry. Quat Sci Rev. 2011;30: 3575–3588. 10.1016/j.quascirev.2011.09.001

[12] de Winter N, Müller I, Kocken I, Thibault N, Ullmann CV, Farnsworth A, et al. First absolute seasonal temperature estimates for greenhouse climate from clumped isotopes in bivalve shells. [Preprint]. 2020. 10.1101/2020.04.15.20064931 32511587 PMC7276014

[13] Fiebig J, Bajnai D, Löffler N, Methner K, Krsnik E, Mulch A, et al. Combined high-precision Δ48 and Δ47 analysis of carbonates. Chem Geol. 2019;522: 186–191. 10.1016/j.chemgeo.2019.05.019

[14] Schöne BR, Radermacher P, Zhang Z, Jacob DE. Crystal fabrics and element impurities (Sr/Ca, Mg/Ca, and Ba/Ca) in shells of *Arctica islandica*—Implications for paleoclimate reconstructions. Palaeogeogr Palaeoclimatol Palaeoecol. 2013;373: 50–59. 10.1016/j.palaeo.2011.05.013

[15] Nishida K, Ishimura T, Suzuki A, Sasaki T. Seasonal changes in the shell microstructure of the bloody clam, *Scapharca broughtonii* (Mollusca: Bivalvia: Arcidae). Palaeogeogr Palaeoclimatol Palaeoecol. 2012;363–364: 99–108. 10.1016/j.palaeo.2012.08.017

[16] Olson IC, Kozdon R, Valley JW, Gilbert PUPA. Mollusk shell nacre ultrastructure correlates with environmental temperature and pressure. J Am Chem Soc. 2012;134: 7351–7358. 10.1021/ja210808s 22313180

[17] Tan Tiu A. Temporal and spatial variation of shell microstructure of *Polydesmoda caroliniana* (Bivalvia: Heterodonta). Am Malacol Bull. 1988;6: 199–206.

[18] Tan Tiu A, Prezant RS. Shell tubules in *Corbicula fluminea* (Bivalvia: heterodonta): functional morphology and microstructure. The Nautilus. 1989;103: 36–39.

[19] Brand U, Morrison JO. Paleoscene #6. Biogeochemistry of fossil marine-invertebrates. Geosci Can. 1987;14: 85–107.

[20] Morrison JO, Brand U. An evaluation of diagenesis and chemostratigraphy of upper cretaceous molluscs from the Canadian Interior Seaway. Chem Geol Isot Geosci Sect. 1988;72: 235–248. 10.1016/0168-9622(88)90027-9

[21] Füllenbach CS, Schöne BR, Mertz-Kraus R. Strontium/lithium ratio in aragonitic shells of *Cerastoderma edule* (Bivalvia)—A new potential temperature proxy for brackish environments. Chem Geol. 2015;417: 341–355. 10.1016/j.chemgeo.2015.10.030

[22] Shirai K, Schöne BR, Miyaji T, Radarmacher P, Krause RA, Tanabe K. Assessment of the mechanism of elemental incorporation into bivalve shells (*Arctica islandica*) based on elemental distribution at the microstructural scale. Geochim Cosmochim Acta. 2014;126: 307–320. 10.1016/j.gca.2013.10.050

[23] Wanamaker AD, Gillikin DP. Strontium, magnesium, and barium incorporation in aragonitic shells of juvenile *Arctica islandica*: Insights from temperature controlled experiments. Chem Geol. 2018; S0009254118300779. 10.1016/j.chemgeo.2018.02.012

[24] Gilbert PUPA, Bergmann KD, Myers CE, Marcus MA, DeVol RT, Sun C-Y, et al. Nacre tablet thickness records formation temperature in modern and fossil shells. Earth Planet Sci Lett. 2017;460: 281–292. 10.1016/j.epsl.2016.11.012

[25] Milano S, Schöne BR, Witbaard R. Changes of shell microstructural characteristics of *Cerastoderma edule* (Bivalvia)—A novel proxy for water temperature. Palaeogeogr Palaeoclimatol Palaeoecol. 2017;465: 395–406. 10.1016/j.palaeo.2015.09.051

[26] Höche N, Peharda M, Walliser EO, Schöne BR. Morphological variations of crossed-lamellar ultrastructures of *Glycymeris bimaculata* (Bivalvia) serve as a marine temperature proxy. Estuar Coast Shelf Sci. 2020;237: 106658. 10.1016/j.ecss.2020.106658

[27] Milano S, Nehrke G, Wanamaker AD, Ballesta-Artero I, Brey T, Schöne BR. The effects of environment on *Arctica islandica* shell formation and architecture. Biogeosciences. 2017;14: 1577–1591. 10.5194/bg-14-1577-2017

[28] Ropes JW, Jones D, Murawski S, Serchuk F, Jearld A. Documentation of annual growth lines in ocean quahogs, *Arctica islandica* Linné. Fish Bull. 1984;82: 1–19.

[29] Trofimova T, Milano S, Andersson C, Bonitz FGW, Schoene BR. Oxygen isotope composition of *Arctica islandica* aragonite in the context of shell architectural organization: Implications for paleoclimate reconstructions. Geochem Geophys Geosystems. 2018;19: 453–470. 10.1002/2017GC007239

[30] Dunca E, Mutvei H, Goransson P, Morth C-M, Schone BR, Whitehouse MJ, et al. Using ocean quahog (*Arctica islandica*) shells to reconstruct palaeoenvironment in Öresund, Kattegat and Skagerrak, Sweden. Int J Earth Sci. 2009; 15. 10.1007/s00531-008-0348-6

[31] Karney GB, Butler PG, Scourse JD, Richardson CA, Lau KH, Czernuszka JT, et al. Identification of growth increments in the shell of the bivalve mollusc *Arctica islandica* using backscattered electron imaging. J Microsc. 2011;241: 29–36. 10.1111/j.1365-2818.2010.03403.x 21118202

[32] Füllenbach CS, Schoene BR, Shirai K, Takahata N, Ishida A, Sano Y. Minute co-variations of Sr/Ca ratios and microstructures in the aragonitic shell of *Cerastoderma edule* (Bivalvia)—Are geochemical variations at the ultra-scale masking potential environmental signals? Geochim Cosmochim Acta. 2017;205: 256–271. 10.1016/j.gca.2017.02.019

[33] Karney GB, Butler PG, Speller S, Scourse JD, Richardson CA, Schröder M, et al. Characterizing the microstructure of *Arctica islandica* shells using NanoSIMS and EBSD. Geochem Geophys Geosystems. 2012;13: n/a-n/a. 10.1029/2011GC003961

[34] Schöne BR. *Arctica islandica* (Bivalvia): A unique paleoenvironmental archive of the northern North Atlantic Ocean. Glob Planet Change. 2013;111: 199–225. 10.1016/j.gloplacha.2013.09.013

[35] Wanamaker AD, Kreutz KJ, Schöne BR, Maasch KA, Pershing AJ, Borns HW, et al. A late Holocene paleo-productivity record in the western Gulf of Maine, USA, inferred from growth histories of the long-lived ocean quahog (*Arctica islandica*). Int J Earth Sci. 2009;98: 19.

[36] Butler PG, Richardson CA, Scourse JD, Wanamaker AD, Shammon TM, Bennell JD. Marine climate in the Irish Sea: analysis of a 489-year marine master chronology derived from growth increments in the shell of the clam *Arctica islandica*. Quat Sci Rev. 2010;29: 1614–1632. 10.1016/j.quascirev.2009.07.010

[37] Lohmann G, Schöne BR. Climate signatures on decadal to interdecadal time scales as obtained from mollusk shells (*Arctica islandica*) from Iceland. Palaeogeogr Palaeoclimatol Palaeoecol. 2013;373: 152–162. 10.1016/j.palaeo.2012.08.006

[38] Walliser EO, Lohmann G, Niezgodzki I, Tütken T, Schöne BR. Response of Central European SST to atmospheric pCO2 forcing during the Oligocene–A combined proxy data and numerical climate model approach. Palaeogeogr Palaeoclimatol Palaeoecol. 2016;459: 552–569. 10.1016/j.palaeo.2016.07.033

[39] Ehrenbaum E. Untersuchungen über die Struktur und Bildung der Schale der in der Kieler Bucht häufig vorkommenden Muscheln. Dissertation, Kiel University. 1884.

[40] Witbaard R, Franken R, Visser B. Growth of juvenile *Arctica islandica* under experimental conditions. Helgoländer Meeresunters. 1997;51: 417–431.

[41] Winter JE. Über den Einfluß der Nahrungskonzentration und anderer Faktoren auf Filtrierleistung und Nahrungsausnutzung der Muscheln Arctica islandica und Modiolus modiolus. Mar Biol. 1969;4: 87–135. 10.1007/BF00347037

[42] Pitts LC, Wallace GT. Lead deposition in the shell of the bivalve, *Mya arenaria*: an indicator of dissolved lead in seawater. Estuar Coast Shelf Sci. 1994;39: 93–104. 10.1006/ecss.1994.1051

[43] Price GD, Pearce NJG. Biomonitoring of pollution by *Cerastoderma edule* from the British Isles: a laser ablation ICP-MS study. Mar Pollut Bull. 1997;34: 1025–1031. 10.1016/S0025-326X(97)00098-2

[44] Markich SJ, Jeffree RA, Burke PT. Freshwater bivalve shells as archival indicators of metal pollution from a copper−uranium mine in tropical Northern Australia. Environ Sci Technol. 2002;36: 821–832. 10.1021/es011066c 11918003

[45] Liehr GA, Zettler ML, Leipe T, Witt G. The ocean quahog *Arctica islandica* L.: a bioindicator for contaminated sediments. Mar Biol. 2005;147: 671–679. 10.1007/s00227-005-1612-y

[46] Jeffree RA, Markich SJ, Lefebvre F, Thellier M, Ripoll C. Shell microlaminations of the freshwater bivalve *Hyridella depressa* as an archival monitor of manganese water concentration: experimental investigation by depth profiling using secondary ion mass spectrometry (SIMS). Experientia. 1995;51: 838–848. 10.1007/BF01922440

[47] Barbin V, Ramseyer K, Elfman M. Biological record of added manganese in seawater: a new efficient tool to mark in vivo growth lines in the oyster species *Crassostrea gigas*. Int J Earth Sci. 2008;97: 193–199. 10.1007/s00531-006-0160-0

[48] Kremling K, Petersen H. The distribution of Mn, Fe, Zn, Cd and Cu in Baltic seawater; a study on the basis of one anchor station. Mar Chem. 1978;6: 155–170. 10.1016/0304-4203(78)90025-7

[49] Kremling K. The behavior of Zn, Cd, Cu, Ni, Co, Fe, and Mn in anoxic Baltic waters. Mar Chem. 1983;13: 87–108. 10.1016/0304-4203(83)90019-1

[50] van de Velde SJ, Hylen A, Kononets M, Marzocchi U, Leermakers M, Choumiline K, et al. Elevated sedimentary removal of Fe, Mn, and trace elements following a transient oxygenation event in the Eastern Gotland Basin, central Baltic Sea. Geochim Cosmochim Acta. 2020;271: 16–32. 10.1016/j.gca.2019.11.034

[51] Bonsdorff E. Zoobenthic diversity-gradients in the Baltic Sea: Continuous post-glacial succession in a stressed ecosystem. J Exp Mar Biol Ecol. 2006;330: 383–391. 10.1016/j.jembe.2005.12.041

[52] Conley DJ, Carstensen J, Aigars J, Axe P, Bonsdorff E, Eremina T, et al. Hypoxia Is Increasing in the Coastal Zone of the Baltic Sea. Environ Sci Technol. 2011;45: 6777–6783. 10.1021/es201212r 21770387 PMC3155394

[53] Hansson D, Gustafsson E. Salinity and hypoxia in the Baltic Sea since A.D. 1500. J Geophys Res Oceans. 2011;116. 10.1029/2010JC006676

[54] Carstensen J, Conley DJ, Bonsdorff E, Gustafsson BG, Hietanen S, Janas U, et al. Hypoxia in the Baltic Sea: biogeochemical cycles, benthic fauna, and management. Ambio. 2014;43: 26–36. 10.1007/s13280-013-0474-7 24414802 PMC3888664

[55] Jochum KP, Weis U, Stoll B, Kuzmin D, Yang Q, Raczek I, et al. Determination of reference values for NIST SRM 610–617 glasses following ISO guidelines. Geostand Geoanalytical Res. 2011;35: 397–429. 10.1111/j.1751-908X.2011.00120.x

[56] Branson O, Fehrenbacher JS, Vetter L, Sadekov AY, Eggins SM, Spero HJ. LAtools: A data analysis package for the reproducible reduction of LA-ICPMS data. Chem Geol. 2019;504: 83–95. 10.1016/j.chemgeo.2018.10.029

[57] Jochum KP, Nohl U, Herwig K, Lammel E, Stoll B, Hoffmann A. GeoReM: A new geochemical database for reference materials and isotopic standards. Geostand Geoanalytical Res. 2007;29: 333–338.

[58] Schöne BR, Dunca E, Fiebig J, Pfeiffer M. Mutvei’s solution: An ideal agent for resolving microgrowth structures of biogenic carbonates. Palaeogeogr Palaeoclimatol Palaeoecol. 2005;228: 149–166. 10.1016/j.palaeo.2005.03.054

[59] Crippa G, Ye F, Malinverno C, Rizzi A. Which is the best method to prepare invertebrate shells for SEM analysis? Testing different techniques on recent and fossil brachiopods. Boll Della Soc Paleontol Ital. 2016;55: 111–125. 10.4435/BSPI.2016.11

[60] Berg S, Kutra D, Kroeger T, Straehle CN, Kausler BX, Haubold C, et al. ilastik: interactive machine learning for (bio)image analysis. Nat Methods. 2019;16: 1226–1232. 10.1038/s41592-019-0582-9 31570887

[61] Schneider CA, Rasband WS, Eliceiri KW. NIH Image to ImageJ: 25 years of image analysis. Nat Methods. 2012;9: 671–675. 10.1038/nmeth.2089 22930834 PMC5554542

[62] Schindelin J, Rueden CT, Hiner MC, Eliceiri KW. The ImageJ ecosystem: An open platform for biomedical image analysis. Mol Reprod Dev. 2015;82: 518–529. 10.1002/mrd.22489 26153368 PMC5428984

[63] Rueden CT, Schindelin J, Hiner MC, DeZonia BE, Walter AE, Arena ET, et al. ImageJ2: ImageJ for the next generation of scientific image data. BMC Bioinformatics. 2017;18: 529. 10.1186/s12859-017-1934-z 29187165 PMC5708080

[64] Geurts P, Irrthum A, Wehenkel L. Supervised learning with decision tree-based methods in computational and systems biology. Mol Biosyst. 2009;5: 1593–1605. 10.1039/b907946g 20023720

[65] Lei L, Seol Y, Jarvis K. Pore-scale visualization of methane hydrate-bearing sediments with micro-CT. Geophys Res Lett. 2018;45: 5417–5426. 10.1029/2018GL078507

[66] Jacob A, Peltz M, Hale S, Enzmann F, Moravcova O, Warr LN, et al. Simulating permeability reduction by clay mineral nanopores in a tight sandstone by combining μXCT and FIB-SEM imaging. Solid Earth Discuss. 2020; 1–28. 10.5194/se-2020-151

[67] Clark GR. Mollusk shell: daily growth lines. Science. 1968;161: 800–802. 10.1126/science.161.3843.800 5663810

[68] Palmer RE. Observations on shell deformities, ultrastructure, and increment formation in the Bay scallop *Argopecten irradians*. Mar Biol. 1980;58: 15–23. 10.1007/BF00386874

[69] Sato S, Chiba T. Structural changes in molluscan community over a 15-year period before and after the 2011 Great East Japan Earthquake and subsequent tsunami around Matsushima Bay, Miyagi Prefecture, Northeastern Japan. PLOS ONE. 2016;11: e0168206. 10.1371/journal.pone.0168206 27936182 PMC5148594

[70] Mejri W, Korchef A, Tlili M, Amor MB. Effects of temperature on precipitation kinetics and microstructure of calcium carbonate in the presence of magnesium and sulphate ions. Desalination Water Treat. 2014;52: 4863–4870. 10.1080/19443994.2013.808813

[71] Carré M, Bentaleb I, Bruguier O, Ordinola E, Barrett NT, Fontugne M. Calcification rate influence on trace element concentrations in aragonitic bivalve shells: evidences and mechanisms. Geochim Cosmochim Acta. 2006;70: 4906–4920. 10.1016/j.gca.2006.07.019

[72] Marin F. The formation and mineralization of mollusk shell. Front Biosci. 2012;S4: 1099–1125. 10.2741/s321 22202112

[73] Milano S, Schöne BR, Wang S, Müller WE. Impact of high pCO2 on shell structure of the bivalve *Cerastoderma edule*. Mar Environ Res. 2016;119: 144–155. 10.1016/j.marenvres.2016.06.002 27285613

[74] Gillooly JF, Brown JH, West GB, Savage VM, Charnov EL. Effects of size and temperature on metabolic rate. Science. 2001;293: 2248–2251. 10.1126/science.1061967 11567137

[75] Shibata M. Tubules found in the arcoid shell. Venus Jpn J Malacol. 1979;38: 48–60. doi: venusjjm.38.1_48

[76] Waller TR. Scanning electron microscopy of shell and mantle in the order Arcoida (Mollusca: Bivalvia). Smithson Contrib Zool. 1980; 1–58. 10.5479/si.00810282.313

[77] Malchus N. Shell tubules in Condylocardiinae (Bivalvia: Carditoidea). J Molluscan Stud. 2010;76: 401–403. 10.1093/mollus/eyq030

[78] Palmer AR. Relative cost of producing skeletal organic matrix versus calcification: evidence from marine gastropods. Mar Biol. 1983;75: 287–292. 10.1007/BF00406014

[79] Bevelander G, Nakahara H. Compartment and envelope formation in the process of biological mineralization. In: Ōmori M, Watabe N, editors. The mechanisms of Biomineralization in animals and Plants. Tokai University Press; 1980. pp. 19–27.

[80] Gotliv B-A, Addadi L, Weiner S. Mollusk shell acidic proteins: in search of individual functions. ChemBioChem. 2003;4: 522–529. 10.1002/cbic.200200548 12794863

[81] Cuif J-P, Dauphin Y, Luquet G, Medjoubi K, Somogyi A, Perez-Huerta A. Revisiting the organic template model through the microstructural study of shell development in *Pinctada margaritifera*, the Polynesian Pearl Oyster. Minerals. 2018;8: 370. 10.3390/min8090370

[82] Xiang L, Kong W, Su J, Liang J, Zhang G, Xie L, et al. Amorphous calcium carbonate precipitation by cellular biomineralization in mantle cell cultures of *Pinctada fucata*. Strack S, editor. PLoS ONE. 2014;9: e113150. 10.1371/journal.pone.0113150 25405357 PMC4236139

[83] Agbaje OBA, Thomas DE, Mclnerney BV, Molloy MP, Jacob DE. Organic macromolecules in shells of *Arctica islandica*: comparison with nacroprismatic bivalve shells. Mar Biol. 2017;164: 208. 10.1007/s00227-017-3238-2

[84] Levi-Kalisman Y, Falini G, Addadi L, Weiner S. Structure of the nacreous organic matrix of a bivalve mollusk shell examined in the hydrated state using cryo-TEM. J Struct Biol. 2001;135: 8–17. 10.1006/jsbi.2001.4372 11562161

[85] Agbaje OBA, Thomas DE, Dominguez JG, Mclnerney BV, Kosnik MA, Jacob DE. Biomacromolecules in bivalve shells with crossed lamellar architecture. J Mater Sci. 2019;54: 4952–4969. 10.1007/s10853-018-3165-8

[86] Grefsrud ES, Dauphin Y, Cuif J-P, Denis A, Strand Ø. Modifications in microstructure of cultured and wild scallop shells (*Pecten maximus*). J Shellfish Res. 2008;27: 633–641. 10.2983/0730-8000(2008)27[633:MIMOCA]2.0.CO;2

[87] Goldstein JI, Newbury DE, Michael JR, Ritchie NW, Scott JHJ, Joy DC. Scanning Electron Microscopy and X-Ray Microanalysis. New York, USA: Springer; 2017.

[88] Soreghan MJ, Francus P. Processing backscattered electron digital images of thin section. In: Francus P, editor. Image analysis, sediments and paleoenvironments. Dordrecht: Springer Netherlands; 2004. pp. 203–225. 10.1023/b:pham.0000032994.36343.02

[89] Füllenbach CS, Schöne BR, Branscheid R. Microstructures in shells of the freshwater gastropod *Viviparus viviparus*: A potential sensor for temperature change? Acta Biomater. 2014;10: 3911–3921. 10.1016/j.actbio.2014.03.030 24704696

[90] Faÿ-Gomord O, Soete J, Davy CA, Janssens N, Troadec D, Cazaux F, et al. Tight chalk: characterization of the 3D pore network by FIB-SEM, towards the understanding of fluid transport. J Pet Sci Eng. 2017;156: 67–74. 10.1016/j.petrol.2017.05.005

[91] Penkman KEH, Kaufman DS, Maddy D, Collins MJ. Closed-system behaviour of the intra-crystalline fraction of amino acids in mollusc shells. Quat Geochronol. 2008;3: 2–25. 10.1016/j.quageo.2007.07.001 19684879 PMC2727006

[92] Krause‐Nehring J, Klügel A, Nehrke G, Brellochs B, Brey T. Impact of sample pretreatment on the measured element concentrations in the bivalve *Arctica islandica*. Geochem Geophys Geosystems. 2011;12. 10.1029/2011GC003630

[93] Nishida K, Nakashima R, Majima R, Hikida Y. Ontogenetic changes in shell microstructures in the cold seep-associated bivalve, *Conchocele bisecta* (Bivalvia: Thyasiridae). Paleontol Res. 2011;15: 193–212. 10.2517/1342-8144-15.4.193

[94] Ballesta-Artero I, Zhao L, Milano S, Mertz-Kraus R, Schöne BR, van der Meer J, et al. Environmental and biological factors influencing trace elemental and microstructural properties of *Arctica islandica* shells. Sci Total Environ. 2018;645: 913–923. 10.1016/j.scitotenv.2018.07.116 30032087

[95] Carter JG. Skeletal biomineralization: patterns, processes, and evolutionary trends. New York: Van Nostrand Reinhold; 1990.

[96] Böhm CF, Demmert B, Harris J, Fey T, Marin F, Wolf SE. Structural commonalities and deviations in the hierarchical organization of crossed-lamellar shells: a case study on the shell of the bivalve *Glycymeris glycymeris*. J Mater Res. 2016;31: 536–546. 10.1557/jmr.2016.46

[97] Agbaje OBA, Wirth R, Morales LFG, Shirai K, Kosnik M, Watanabe T, et al. Architecture of crossed-lamellar bivalve shells: the southern giant clam (*Tridacna derasa*, Röding, 1798). R Soc Open Sci. 2017;4: 170622. 10.1098/rsos.170622 28989765 PMC5627105

[98] Carter JG, Harries PJ, Malchus N, Sartori AF, Anderson LC, Bieler R, et al. Part N: Illustrated glossary of the Bivalvia. Treatise on Invertebrate Paleontology no 48, Revised. Kansas: University of Kansas; 2012. pp. 1–209. 10.17161/to.v0i0.4322

