# Supplementary material for: Temperature-induced microstructural changes in shells of laboratory-grown Arctica islandica (Bivalvia)
Source: PLoS One. 2021 Feb 26;16(2):e0247968. doi: 10.1371/journal.pone.0247968 (PMC7909638; doi:10.1371/journal.pone.0247968)
Supplement: S1 File — Results of experiments to identify the suitable preparation method and three-dimensional model of the sample topography in SEM. (DOCX) [file pone.0247968.s003.docx]

# S1 File. Sample preparation for morphometric analyses.

Bivalve shells are typically immersed in acids or oxidation agents [1–3] to reveal microstructures and their individual constituents (i.e., BMUs), which are otherwise not distinguishable in SEM. The choice of the chemical agent, the concentration and the duration of immersion determine the degree of etching of the individual BMUs and the overall sample appearance under the SEM. For automated microstructure morphometry, an optimal preparation method should I) outline the boundaries of individual objects of interest as accurately and uniformly as possible, II) keep the topographical relief (= gray value contrast) at a minimum and III) retain the pristine shapes of the individual components to be measured.

In preparation for this study, we tested different acids (HCl, HCOOH) and oxidation agents (NaOCl, H_2_O_2_) in various concentrations and for various immersion times (results summarized in Fig A). Acid treatments inevitably resulted in microscale dissolution of the individual BMUs, which substantially altered their shape and generated strong topographical relief. In addition, incomplete removal of the organic matrix results only in a partial separation of adjoining BMUs. Acid-based shell preparation was therefore not suitable for automatic BMU size measurements. Weak oxidation agents (NaOCl), in contrast, did not affect the BMU morphology. However, treatment with NaOCl did not completely remove the organic matrices and, thus, did not resolve the BMU boundaries. Hydrogen peroxide turned out to be the most suitable preparation agent, because it retained the polished state of the sample surface (low topographical relief; Fig B), removed the superficial organic components thoroughly, and only slightly affected the BMU edges (Figs 2-4 in the main text). A concentration of 10.5% at an immersion time of 20 minutes provided the most distinct results in the studied bivalve species (*A. islandica*).


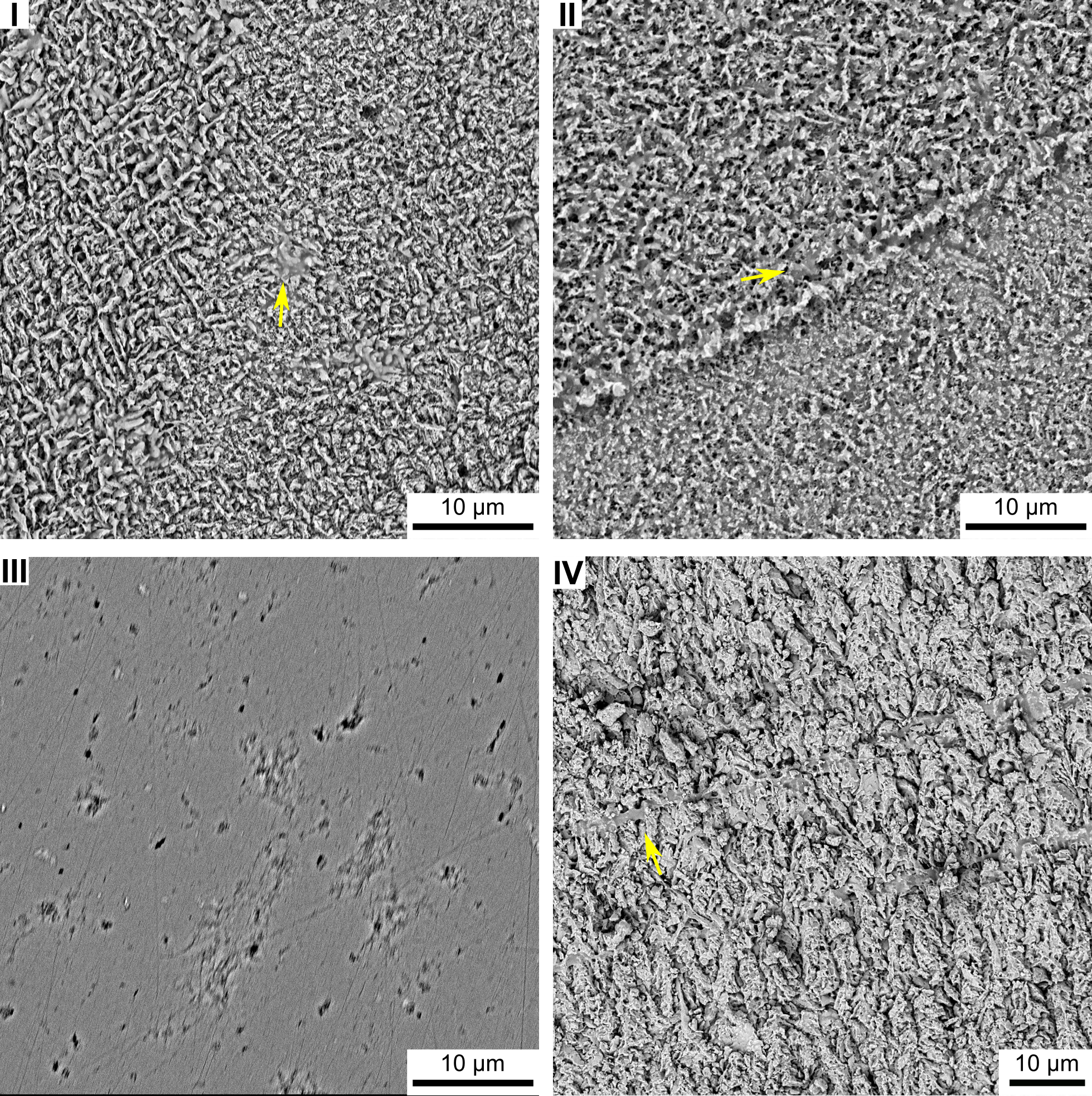


**Fig. A. SEM micrographs of shell slabs of *A. islandica* after various chemical treatments.**

(I) Immersion in 8 % HCl for one second generated a strong topographical relief (= highly variable backscatter intensity) and only incompletely removed interstitial organic components (yellow arrow). (II) Treatment with weaker acids at lower concentration (0.1 % formic acid for 20 minutes) led to partial dissolution of the mineral phase and only marginally removed the organic matrices. (III) Weak oxidation agents (12 % NaOCl for 20 minutes) left the sample surface substantially unaltered. (IV) Immersion in a mixture of 1 % HCl and 5 % commercial bleach for 20 minutes carved out the biomineral morphology reasonably well, but still left behind organic inclusions. Loose mineral grains were scattered on the surface. All samples were sputter-coated with 5 nm Pt and imaged at 10 keV.


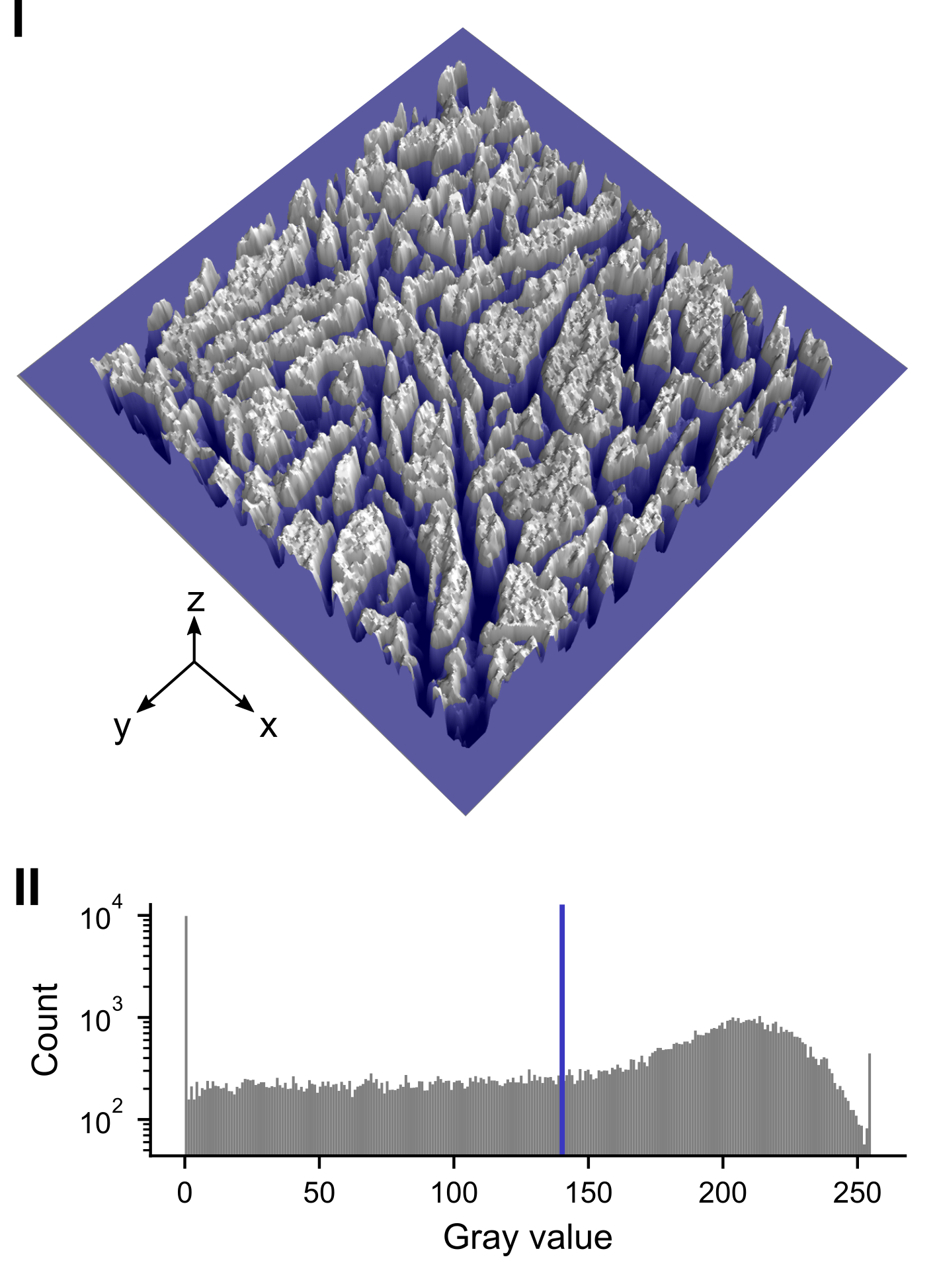


**Fig. B. Three-dimensional visualization of a polished and oxidized shell slab surface.**

(I) The gray value of each pixel of a SEM image was used as a height reference. Biominerals (= bright gray values) appeared as hills, depressions and voids (= dark gray values and black) as valleys. The blue plane represents the average gray value of the image (z = 140), which was used as a threshold value to assess the spatial coverage of the mineral phase in the SEM images. Model was generated using the 3D visualizer mayavi [4]. (II) Gray value histogram of the SEM image used in the 3D visualization. Threshold value for BMU coverage analysis is indicated as vertical blue line.

# References

1. Crippa G, Ye F, Malinverno C, Rizzi A. Which is the best method to prepare invertebrate shells for SEM analysis? Testing different techniques on recent and fossil brachiopods. Boll Della Soc Paleontol Ital. 2016;55: 111–125. doi:10.4435/BSPI.2016.11

2. Milano S, Schöne BR, Witbaard R. Changes of shell microstructural characteristics of *Cerastoderma edule* (Bivalvia) — A novel proxy for water temperature. Palaeogeogr Palaeoclimatol Palaeoecol. 2017;465: 395–406. doi:10.1016/j.palaeo.2015.09.051

3. Dunca E, Mutvei H, Goransson P, Morth C-M, Schone BR, Whitehouse MJ, et al. Using ocean quahog (*Arctica islandica*) shells to reconstruct palaeoenvironment in Öresund, Kattegat and Skagerrak, Sweden. Int J Earth Sci. 2009; 15. doi:10.1007/s00531-008-0348-6

4. Ramachandran P, Varoquaux G. Mayavi: 3D visualization of scientific data. Comput Sci Eng. 2011;13: 40–51. doi:10.1109/MCSE.2011.35
