# Supplementary material for: Temperature-induced microstructural changes in shells of laboratory-grown Arctica islandica (Bivalvia)
Source: PLoS One. 2021 Feb 26;16(2):e0247968. doi: 10.1371/journal.pone.0247968 (PMC7909638; doi:10.1371/journal.pone.0247968)
Supplement: S2 File — Information on the selection of a suitable analysis threshold and justification for the choice of linear and exponential models, respectively. (DOCX) [file pone.0247968.s004.docx]

# S2 File. Statistical analyses of morphometric data.

Since the temperature dependence of BMU and pore size data was most evident in the largest values of the respective datasets, small values were filtered out by applying a threshold. The suitable threshold value was determined by calculating linear regressions for increasingly larger sample sets incorporating the *n* largest BMU and pore size values, respectively (Fig A). The slope of regression lines for both BMU and pore size decreased with increasing sample size. Correlation coefficients (r), coefficients of determination (r²) and standard errors of the models attained maximum values at the lowest sample sizes and asymptotically decreased with increasing *n*. All computed regressions (except that for *n* = 1) were statistically significant (p < 0.05). As a compromise between strong predictive power (i.e., high r, r²) of the model and an adequate sample size, the 15 largest values of each temperature setting were incorporated in the regression analyses.

To determine which type of regression model fits the data, we calculated exponential and linear regressions for each microstructural parameter measured in the study, i.e., BMU size and coverage and pore size (Fig B). In case of BMU size and coverage, the two models exhibited only marginal differences (Fig B I+II). Hence, the more simple linear models were used for BMU size and coverage in the manuscript. In case of pore size, however, linear regressions predicted negative BMU size values for temperatures below ca. 2 °C, which is physically impossible (Figs A II, B III). Use of the exponential regression model prevented this effect. In addition, the residuals of the exponential model were more randomly distributed than those of the linear model, eliminating a systematic prediction bias that otherwise would have been introduced (Fig B VI).


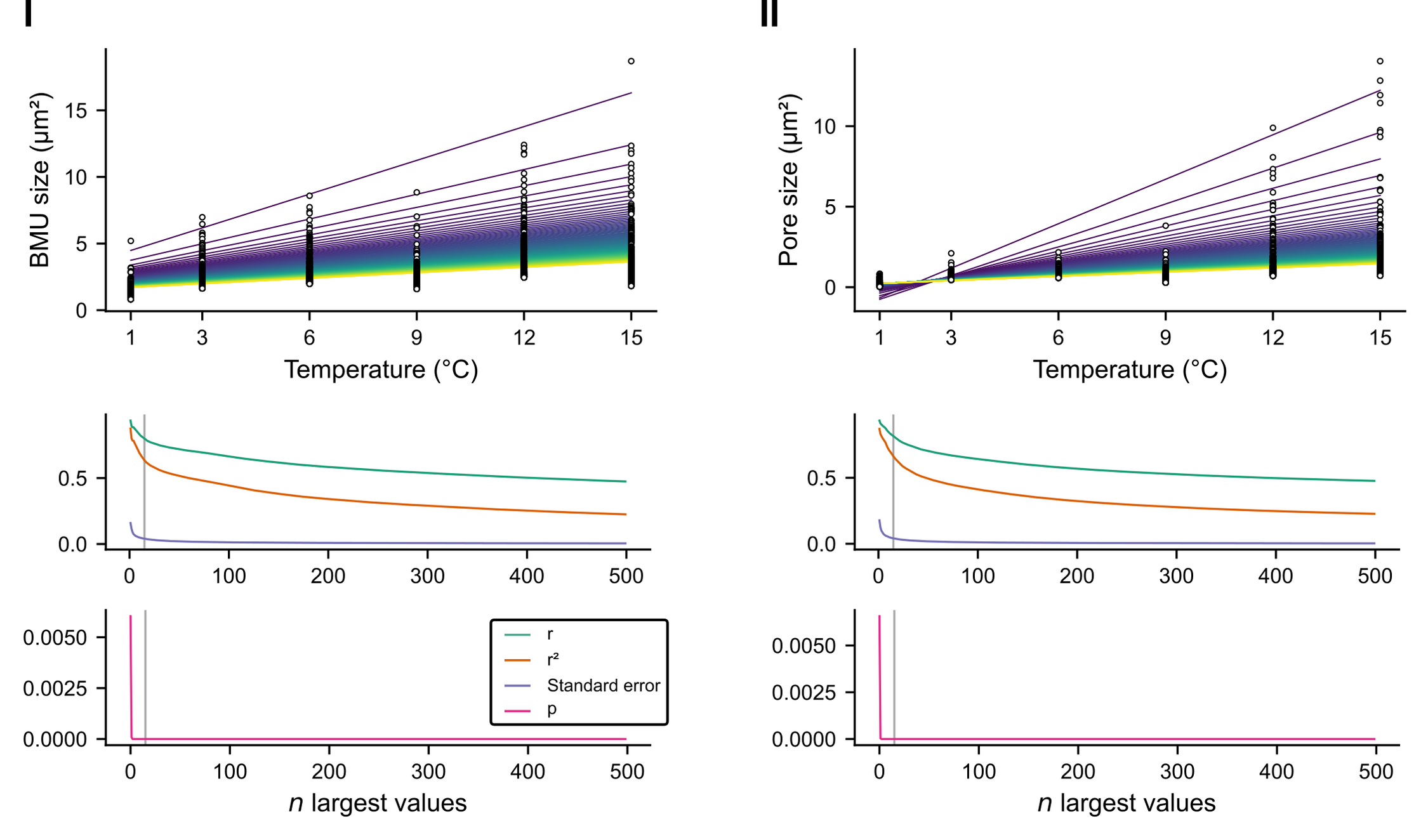
**Fig. A. Linear models and their corresponding statistics for (I) BMU size and (II) pore size computed for different thresholds.**

Top row: Linear regressions were computed for the *n* (1–500, step size = 5) largest BMUs and pores of each temperature setting. Each line represents one regression; Line color shifts from purple to yellow with increasing *n*. White circles represent the 500 largest values of each temperature setting. Bottom rows: Correlation coefficients (r), coefficients of determination (r²), standard errors and p-values for regressions computed with increasing *n*. Threshold used in this study (*n* = 15) is indicated as vertical gray line.


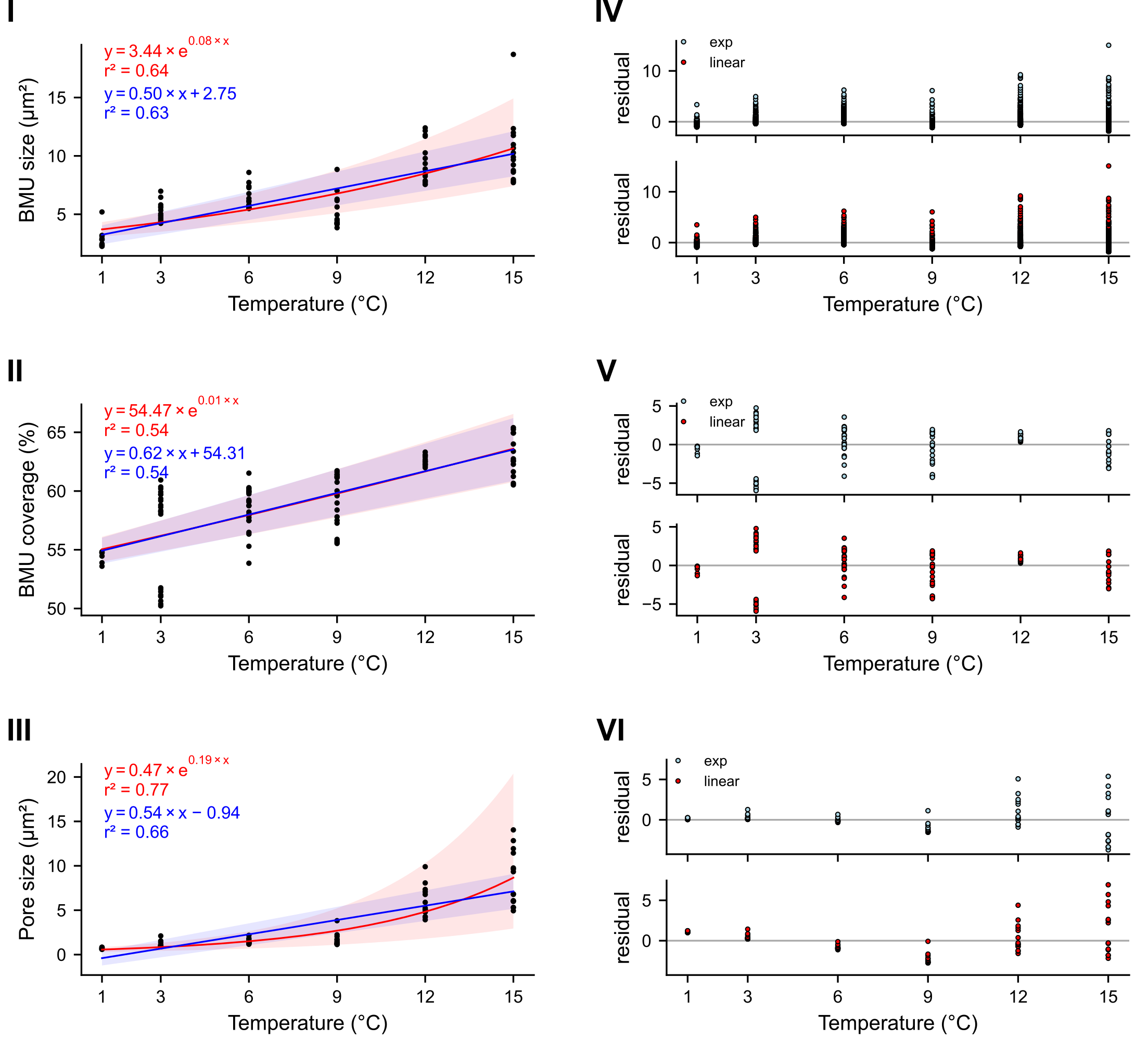
**Fig. B. Comparison of exponential and linear models fitted to each of the measured microstructural parameters.**

(I-III) Exponential and linear models fitted to microstructural data. In case of the size of the 15 largest BMUs (I) and the BMU coverage (II), models exhibited a near complete overlap. In case of the size of the 15 largest pores (III), in contrast, models overlapped to a lesser degree. (IV-VI) Residuals (y – y_predicted_) of the model functions presented in A-C. In the case of BMU size (IV) and coverage (V), residuals of both models were randomly distributed and showed only minor differences in their values. Residuals of pore size (VI), in contrast, displayed non-random distributions for the linear model, leading to a bias toward large pore sizes at low and high temperatures (1–3 and 12–15 °C), and toward small values in the intermediate temperature range (6–9 °C). An exponential fit successfully eliminated this effect and produced more randomly distributed residuals with a smaller average value.
