# Supplementary material for: Temperature-induced microstructural changes in shells of laboratory-grown Arctica islandica (Bivalvia)
Source: PLoS One. 2021 Feb 26;16(2):e0247968. doi: 10.1371/journal.pone.0247968 (PMC7909638; doi:10.1371/journal.pone.0247968)
Supplement: S3 File — (DOCX) [file pone.0247968.s005.docx]

# S3 File. Evaluation of the image segmentation process.

The reproducibility of machine learning–based image processing was assessed by comparing the segmentation results of four SEM images to their manually generated ‘ground truth’ or ‘gold standard’ segmentations (Fig A I-III). Of the four images, two were taken in shell portions grown under controlled temperature conditions (1 and 15 °C, 34.9 × 34.9 µm) and two images (17.45 × 17.45 µm) in shell portions grown in the Baltic Sea under natural conditions, which show CA and FCCL microstructures. The binary images resulting from manual and automated workflows were compared visually and by means of classification metrics, namely the Jaccard index (JI; [1]) and the variation of information (VI; [2]). The JI represents a similarity measure between two sets ranging from 0 to 100 %. The VI assesses the entropy of both under-segmentation and over-segmentation. The entropy of under-segmentation refers to the uncertainty at which BMUs were interpreted as one entity, despite representing multiple ones (false merges; x-axis in Fig A IV). Over-segmentation, in turn, describes splitting a BMU into multiple ones, despite representing a single entity in the manually generated ‘ground truth’ segmentation (false splits; y-axis in Fig A IV). The scale of this metric is standardized with smaller values implying better segmentation performance. Zero indicates no uncertainty and identity with the ground truth, whereas a value of 1 implies a chance of 50 % to correctly split or merge an element (= BMU) when two phases are to be considered (= BMU and inter-crystalline space).

For images of shell portions grown under controlled temperature conditions, comparisons of manual and automated image segmentations (Fig A) yielded JI values of 84.8 and 83 %, respectively. The entropy of over- and under-segmentation were relatively small (0.46–0.50 and 0.46–0.47, respectively). Comparable values were attained for automatic segmentation processes of similar studies [3,4], demonstrating the reliability of the machine-learning based method applied here. Microstructures grown in the Baltic Sea yielded JI values of 85.7 % and 90.9 % for CA and FCCL microstructures, respectively. VI values were even lower than in the temperature-controlled portions, attaining values of 0.41 and 0.45 for CA, and 0.22 and 0.28 for FCCL, respectively (Fig A IV). These results suggest that BMUs of environmentally grown shell portions can also be faithfully determined by the automated image segmentation process. FCCL microstructures can be detected with even more confidence given their lower VI values compared to all other investigated images. This might be due to the increased co-alignment of adjoining BMUs and the increased spatial regularity.


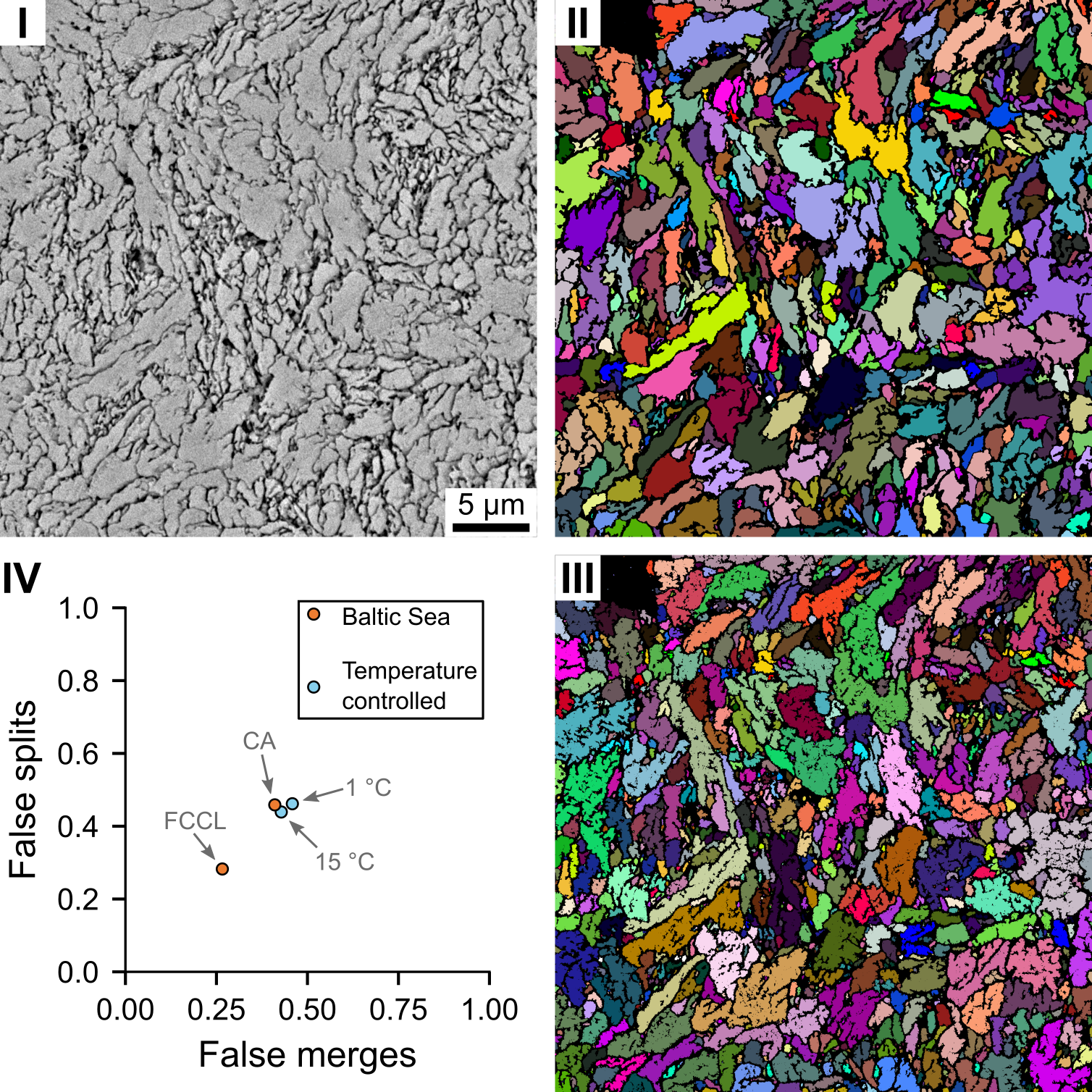


**Fig. A. Comparison between manually and automatically segmented images.**

(I) SEM image of specimen Nioz-TC-15-A1R used for BMU size analysis. (II) Manual segmentation (‘ground truth’) of the SEM image. Individual BMUs are displayed in various colors. (III) Machine learning–based segmentation as used in the study. (IV) Plot of the VI [2]. The x-axis represents the entropy of false merges, whereas the y-axis represents the entropy of false splits. Smaller values imply less uncertainty, i.e., better segmentation performance (0 = identical to the ‘ground truth’).

# References

1. Jaccard P. The distribution of the flora in the alpine zone. New Phytol. 1912;11: 37–50. doi:10.1111/j.1469-8137.1912.tb05611.x

2. Meilă M. Comparing clusterings—an information based distance. J Multivar Anal. 2007;98: 873–895. doi:10.1016/j.jmva.2006.11.013

3. Höche N, Peharda M, Walliser EO, Schöne BR. Morphological variations of crossed-lamellar ultrastructures of *Glycymeris bimaculata* (Bivalvia) serve as a marine temperature proxy. Estuar Coast Shelf Sci. 2020;237: 106658. doi:10.1016/j.ecss.2020.106658

4. Ilett M, Wills J, Rees P, Sharma S, Micklethwaite S, Brown A, et al. Application of automated electron microscopy imaging and machine learning to characterise and quantify nanoparticle dispersion in aqueous media. J Microsc. 2020;279: 177–184. doi:https://doi.org/10.1111/jmi.12853
